# Presenting Features Audiovisually Improves Working Memory for Bindings

**DOI:** 10.5334/joc.481

**Published:** 2026-01-27

**Authors:** Nora Turoman, Elodie Walter, Anaë Motz, Laura-Isabelle Klatt

**Affiliations:** 1 Faculty of Psychology and Educational Sciences, University of Geneva, Geneva, Switzerland; 2 Center for the Interdisciplinary Study of Gerontology and Vulnerabilities, University of Geneva, Carouge, Switzerland; 3 Leibniz Research Centre for Working Environment and Human Factors, Dortmund, Germany

**Keywords:** multisensory, audiovisual, cross-modal, binding, working memory, bimodal advantage

## Abstract

It has long been known that presenting information to multiple senses at a time (e.g., audiovisual presentation as opposed to only visual or auditory) improves later recall of said information – an effect known as the bimodal advantage. Surprisingly however, evidence for this has come only from studies employing free and serial recall, where the identity of an object is recalled, but not in cued recall, where one object feature is recalled when another one is cued. This is despite both tasks requiring binding features into an object in working memory (WM) – our brain’s capacity-limited system for temporarily maintaining information for the purpose of achieving behavioral goals. The present study investigated this discrepancy across a series of four experiments. Contrary to the literature, and despite near-identical task settings, we found evidence in favor of a bimodal advantage across multiple experiments. Moreover, our results suggest that this advantage mainly arises from perceptual processes at encoding rather than from storage in an audiovisual fashion in WM. Finally, a primarily perceptually-based process, the bimodal advantage appears to be sensitive to the characteristics of the cue feature (i.e., its presentation modality). In sum, our results shed light on the mechanism of the bimodal advantage, now robustly detected in cued recall tasks, furthering our understanding of the relationship between perception and WM. Results are discussed in relation to prior studies that did not find a bimodal advantage, potential mechanisms underlying the effect, and the broader framework of the multicomponent model of WM.

Our external environment is rife with information that presents itself in various sensory formats, often stimulating multiple senses at a time. It has long been known that multisensory information is processed differently than the simple sum of its unisensory components or each of these components individually. For instance, early work has shown that the integration of information across vision and audition can create completely unique perceptual experiences (see e.g., the McGurk effect, McGurk & MacDonald, 1976). Another classic line of work has shown that neurons in the cat superior colliculus that respond to several sensory inputs, react to multisensory inputs in ways that are nonlinearly higher or lower than the simple addition or subtraction of the responses to their component unisensory inputs (Meredith et al., 1987; Meredith & Stein, 1983, 1986). These enhanced and unique neural signatures afford multisensory stimuli higher perceptual salience compared to unisensory ones (Stein & Stanford, 2008), which can make object detection faster (e.g., Doyle & Snowden, 2001; Schnupp et al., 2005; Van der Burg et al., 2008). In a famous example, Van der Burg et al. (2008) have shown that a briefly presented uninformative pure tone can speed up search for a visually presented target amid multiple similar visual distractors. However, apart from enhanced salience, multisensory inputs are also imbued with more information than unisensory inputs. For example, understanding the content of a conversation is much easier when we have access both to the visual input (the interlocuter’s lip movements and gestures) and the auditory input (the sound of the interlocuter’s speech) than if we only had access to one of these inputs. In the domain of working memory specifically, our brain’s limited-capacity system for temporary information maintenance in service of behavioral goals (Baddeley & Hitch, 1974; Cowan, 1998; Miller, 1956), an information advantage of multisensory over unisensory inputs has long been posited to be the mechanism for superior recall of stimuli presented audiovisually compared to stimuli only presented visually or aurally (i.e., the so-called bimodal advantage; Thompson & Paivio, 1994, see also Penney, 1989). For example, proponents of the feature model of working memory have argued that this bimodal advantage stems from audiovisually-presented information resulting in representations that are richer in physical features (Nairne, 1990; Neath, 2000). Indeed, apart from object detection, recognition, and attention (for reviews see e.g., Calvert et al., 2004; Murray & Wallace, 2011; Stein, 2012), cognitive processes like working memory have been shown to benefit from multisensory (specifically, audiovisual) stimulus presentation, as we elaborate below. On a similar note, improvements in working memory performance are typically observed when participants produce or actively generate verbal labels during encoding (MacLeod et al., 2010; Whitridge et al. 2025; Overkott, Souza, & Morey, 2023). This so-called *production effect* is thought to originate because production evokes additional memory traces (such as auditory features or a motor code) that are bound to the encoded stimulus (Fawcett, 2013). This additional production trace is assumed to enhance the distinctiveness of such items (MacLeod et al., 2010, MacLeod et al., 2022). In addition, deeper processing or attentional increases may play a crucial role in explaining the advantage (Whitridge et al., 2025). Together, these findings corroborate the notion that presentation format and processes engaged at encoding critically influence the fidelity of working memory.

In continuous recognition studies, where participants had to report whether an item matched previously encountered memoranda or not, audiovisually-presented stimuli were typically remembered better (e.g., Lehmann & Murray, 2005; Matusz et al., 2015; Murray et al., 2004; Thelen et al., 2012, 2015; Xie et al., 2017, 2019, 2021). However, there has been contradicting evidence (e.g., Cohen et al., 2009; Nyberg et al., 2000). Moreover, in a recent study, Pecher and Zeelenberg (2022) corrected an imbalance in the trial numbers of the different modality conditions and found that the superior effect of audiovisual memoranda on recognition had disappeared. However, in recall studies, where participants had to remember and (verbally) report the items they previously encountered, audiovisually-presented objects were always remembered better than either visually- or aurally-presented memoranda (D. E. Broadbent, 1956; Delogu et al., 2009; Goolkasian & Foos, 2005; Hede, 1980; Lewandowski & Kobus, 1993; Martin, 1980; Thompson & Paivio, 1994). Most of the recall studies above have employed verbalizable stimuli and required a verbal response, meaning that representing memoranda in a verbal way was the most adaptive for the task. Assuming that memoranda were represented verbally regardless of presentation format, observing a bimodal advantage here suggests that working memory performance is affected by the presentation format of memoranda. The potential implications of this are important since working memory plays a crucial role in numerous daily tasks: we rely on it during conversations (e.g., to keep in mind what the interlocutor said while we think of a response), while working (e.g., to keep track of what we want to say while composing an email) or when running errands (e.g., to maintain the items on a just-seen shopping list while we navigate to the correct isle). Thus, presenting information in a multisensory (or at least bimodal, audiovisual) format might help boost our working memory in situations where it is highly important for achieving good outcomes, for example in learning (H. J. Broadbent et al., 2018; Brunyé et al., 2008; Lee & Shin, 2012; Pleisch et al., 2019). However, while these findings hint at the promise of multisensory enhancement for working memory, the precise mechanisms underlying these benefits are still unclear.

A key unresolved issue is that the relationship between how memoranda are *presented* and how they are *represented* in working memory in their physical absence is not fully understood. For instance, the above recall studies which have consistently found a bimodal audiovisual advantage all involve *free and serial recall* (Delogu et al., 2009; Goolkasian & Foos, 2005; Lewandowski & Kobus, 1993), where the identity of an object is recalled. Namely, after being shown a list of objects with different presentation modalities, participants are asked what objects they encountered. However, in *cued recall tasks* (Cinar et al., 2025, Guazzo et al., 2020), where one object feature (e.g., color) is used as a cue to recall another feature (e.g., its shape), or recognition tasks which explicitly require cross-modal binding (Allen et al., 2009), such a bimodal advantage has not been found. In cued recall tasks (Cinar et al., 2025, Guazzo et al. 2020), after being shown a list of colored shapes, participants were shown one feature (e.g., a shape), and had to report its associated feature (e.g., its color). What makes the discrepancy between free/serial recall and cued recall tasks especially puzzling is that both tasks should rest on the same mechanism for treating audiovisual memoranda. That is, both tasks require binding auditory and visual features together, and thus, in both tasks should audiovisual memoranda be informationally richer. To address this issue, a study is needed that investigates why such discrepancies exist, and what that implies for working memory in sensorily rich environments.

## The present study

**Figure 1:**
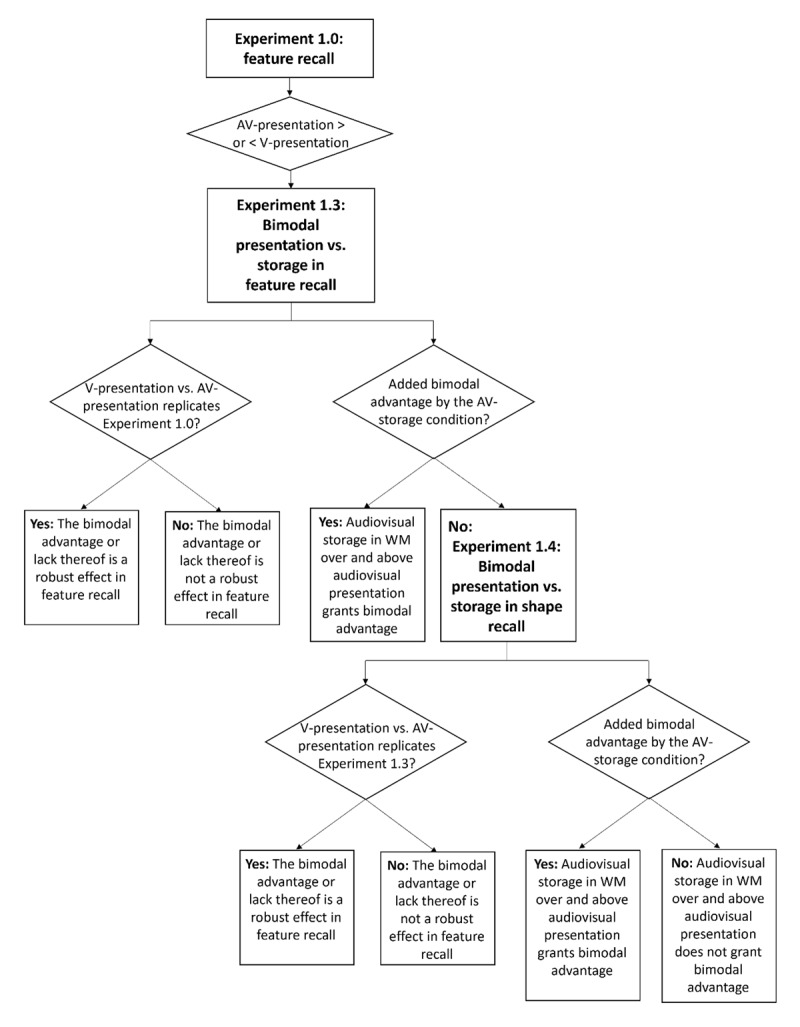
A flowchart of the preregistered experiments conducted as part of the current study following the initial experiment (Experiment 1.0). Experiments were carried out sequentially, top to bottom, depending on the decision taken after the preceding experiment – Experiments and results are shown in square boxes, and decision points are shown in diamond boxes. AV and V stand for audiovisual and visual, respectively.

Two main questions emerge at this stage: 1) why is there a bimodal advantage in one task, but not the other, if they both involve the same mechanism, and 2) how is information of different presentation formats represented in working memory? To address these questions, the present study began with a replication of the most recent cued recall study that did not find a bimodal advantage, namely Cinar and colleagues (2025).^1^ Based on the results of this initial experiment (Experiment 1.0), we followed a pre-registered decision tree that outlined several potential sequences of experiments (full flowchart in Supplemental Figure 1 in the Supplementary materials, flowchart of experiments conducted in this study in Figure 1 below). The design, methods, and hypotheses for each of these experiments were preregistered on the Open Science Framework (OSF), at the following link: https://osf.io/bfpre. Throughout the manuscript, we use the term *bimodal* to denote audiovisual stimulus presentation, in which each modality contributes unique information about a given item (e.g., a visual shape paired with a color presented auditorily), rather than redundant or semantically matched cues (e.g., a written word with its spoken form, or an image of a dog and the sound of a dog bark). *This was done to prevent participants from effectively encoding only one feature and allowed us to implement a cued recall test, in which one feature is used to prompt recall of the associated feature*.

As a general soundness check, to ensure our task is well-posed to measure working memory recall performance, we expected recall accuracy to increase with serial position, consistent with classic findings in the working memory literature (Murdock, 1962). The key hypotheses that guided the sequence of experiments are presented below.

Experiment 1.0 revealed a bimodal advantage, contrary to Cinar et al. (2025), where no such advantage was observed. As a result, we did not proceed with Experiment 1.1, which was designed to test whether the lack of a bimodal advantage arises from the fact that audiovisually and visually presented items are represented in a common code (see Supplementary Material for details on this part of the decision tree).

Instead, in line with our preregistration, we moved to Experiment 1.3 to investigate the source of the observed bimodal advantage. Specifically, we asked: does the bimodal advantage arise from *presenting* information to two sensory modalities, or from subsequentially *storing* information in two modalities within working memory. The former hypothesis assumes that multimodal input alone is sufficient for a bimodal advantage to occur – even if the information is later recoded into a single (e.g., verbal) code, irrespective of the presentation format. The second posits that a multisensory representation is formed in working memory, either by preserving the dual code or by integrating them into a unified, representation. In Experiment 1.3, we kept the visual and audiovisual presentation conditions identical to Experiment 1.0 and added a third audiovisual storage condition. This new condition included pure tones as auditory stimulus material and as the cue dimension (i.e., recalling the shape based on a pitch cue), to promote bimodal storage and minimize the possibility of recoding into a different representational format (e.g., verbal or visual). Contrasting the original *visual* and *audiovisual presentation* condition, we expected to observe the same direction of effect (bimodal advantage) as in Experiment 1.0, which would indicate robustness of the effect. Additionally, if the *audiovisual storage* condition would grant benefits for recall over and above the visual presentation and audiovisual presentation condition, this would show that bimodal *storage* is a key driver of the bimodal advantage. If not, then bimodal storage may not be necessary for producing a bimodal advantage, over and above bimodal presentation.

However, the audiovisual storage condition in Experiment 1.3 differed from the other two in terms of the recalled feature (i.e., recalling shape versus color). While this difference arose intentionally (see Experiment 1.3 Methods for details), it introduces a potential confound. Thus, to ensure that the outcomes would be due to bimodal presentation versus storage, and not other spurious differences between conditions, we harmonized the recall demands (i.e., always recalling shape) in a subsequent Experiment 1.4. This allowed a cleaner test of the distinction between bimodal presentation and bimodal storage, free from task-structure differences. The hypotheses remained the same as in Experiment 1.3, now without the potential caveats. Unexpected results from Experiment 1.4 prompted a final follow-up (Experiment 1.5), which was preregistered only after completing the first three experiments. Experiment 1.5 aimed at differentiating the effects of different cue formats (verbal cue vs. visual cue) on the bimodal advantage.

## Experiment 1.0

### Methods

A detailed description of the sampling plan, task design, and planned analyses for each successive experiment is available in the preregistration for this project: https://osf.io/bfpre. Here, we briefly recapitulate this information and describe methodological aspects that are not described in the preregistration.

#### Participants

A total of 30 young adult undergraduate students at the University of Geneva participated in Experiment 1.0 in exchange for course credits. Two did not pass the following preregistered exclusion criteria: 1) completing the entire experiment, 2) having an accuracy equal to or greater than 25% (i.e., chance-level, since there were 4 possible objects to remember on each trial) in each one of the Binding conditions. Thus, the final sample consisted of 28 participants (age range 19 – 24, mean age 21, 4 males). Participants gave informed consent before participating in the study. All research procedures were approved by the University of Geneva Ethical Commission (approval code: CUREG-2024-02-20).

#### Design and stimuli

Experiment 1.0 had a 3 × 4 repeated-measures factorial design with within-subject factors Binding Condition (Unitized Object-Visual [UO-V], Spatially Separated Object-Visual [SS-V], Unitized Object-AudioVisual [UO-AV]) and Serial position (1, 2, 3, 4). Visual stimuli were modelled after those used by Cinar et al. (2025, see also Allen et al., 2009, Baddeley et al., 2010; Karlsen et al., 2010), and consisted of eight colors (red, green, blue, turquoise, pink, yellow, orange, purple), which could be presented in the form of eight shapes in the UO-V condition (circle, diamond, cross, arch, chevron, star, flag, triangle), in the form of a neutral ‘blob’ shape similar to the one in Allen and colleagues (2009) in the SS-V condition, or as spoken words accompanying the above eight shapes in the UO-AV condition. However, unlike in the studies by Allen and colleagues (e.g., Allen et al., 2006, 2009; Baddeley et al., 2010; Karlsen et al., 2010), instead of the colors black and grey, we used the colors orange and pink, respectively, in order to harmonize color choices between this and any other possible subsequent experiment. Specifically, if Experiment 1.0 had been followed up by Experiment 1.1, black and grey would not make sense to add to a color spectrum slider, and thus orange and pink were chosen, as they seemed similarly as common and recognizable as the other colors in the set. Each visual stimulus was presented on a white background and subtended approximately 2.86 degrees. The HSL values of the eight colors were as follows: red (H: 0, S: 255, L: 128), green (70, 255, 128), blue (159, 255, 128), turquoise (122, 255, 128), pink (221, 255, 128), yellow (42, 255, 128), orange (25, 255, 128), purple (193, 255, 128). Saturation and luminance were kept constant, while the hues were chosen such that they fall into canonical color boundaries on the HSL color wheel (see e.g., https://tympanus.net/codrops/css_reference/hsl/).^2^ Auditory stimuli were recordings (digitized at 44,100 Hz) of a female native French speaker uttering the names of the above eight colors in French. Due to natural variation in spoken word durations, the length of the audio recordings ranged between approximately 570 and 720 ms. These stimuli were presented binaurally through over-ear headphones (Sennheiser HD 25-1 II). The playback volume on each testing computer was set to a volume of 13 (on a 0–100 scale), as this was revealed to be a noticeable yet comfortable volume with our specific hardware setup during pilot testing. Probe stimuli were black outlines, with no color fill, of the above eight shapes. Color-shape combinations were randomly determined per participant, per trial. All stimuli used in Experiment 1.0 are available in the Experiment section of the OSF repository for this project (https://osf.io/fg3wb/).

#### Task and procedure

**Figure 2:**
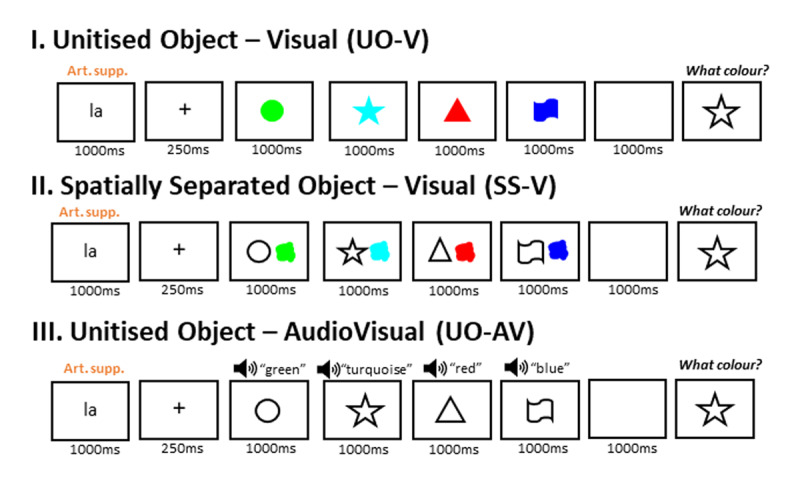
Schematic representation of a trial for each Binding condition in Experiment 1.0, closely following the paradigm of Cinar et al., 2025 in their no-prioritization condition. The same shape-color combinations are used here as an example, but in the actual task, pairings were chosen at random. The textbox that participants saw during the probe is omitted from the figure.

Participants were seated approximately 90 cm from a 24-inch LCD monitors (144-Hz refresh rate, Philips 242G5DJEB) in a group testing room equipped with 6 computers. The room was kept quiet throughout testing, and participants wore over-ear headphones (Sennheiser HD 25-1 II) to minimize ambient noise and ensure consistent auditory presentation. The experiment began with a general set of instructions, explaining the memorization aspect of the task, showing the eight possible shapes and colors, along with their verbal labels, and instructing participants to respond as quickly and accurately as possible. Next, participants were directed to one of three Binding conditions, the presentation of which was counterbalanced across participants. For each condition, to maximize task procedure comprehension, participants first saw a slowed down demonstration of the trial sequence. The latter was accompanied by instructional text on the bottom of the screen, explaining what they needed to do in each stage of a trial. After this, 5 practice trials were given at normal speed, followed by 60 experimental trials. The stages that made up the practice and experimental trials were the following (see also Figure 2): a 1000 ms instruction screen, a 250 ms fixation screen, four successive 1000 ms item displays with 250 ms blank screen breaks in between, a 1000 ms delay screen, and finally a probe screen that ended when the participant finished typing their response. The instruction screen displayed the syllable ‘la’ in black Open Sans font letters on a white background, instructing participants to continuously utter ‘la la la la’ until the probe screen. Such articulatory suppression is a common (e.g., Allen et al., 2006; Baddeley et al., 1975; Morey & Cowan, 2005), and successful (Saito, 1997) form of inhibiting, or at least dampening, verbal coding and maintenance of memoranda. The fixation screen presented a black fixation cross in the center of the screen on a white background. Next, four to-be-remembered items were presented in succession. In the UO-V condition, items consisted of color-shape combinations presented as visually unitized objects in the center of the screen. In the SS-V condition, the color-shape combination items were presented as visually separate entities, with a colored blob shown next to its corresponding black outline shape with no color fill (with colored blobs always on the right). The stimuli were presented at an eccentricity of 1.5° and with ~3° distance between the two. Meanwhile, in the UO-AV condition, the color-shape combination items were presented as a black outline shape with no color fill accompanied by a color-word auditory stimulus. The onset of auditory and visual stimuli was always temporally synchronous. Given the naturally varying length of the verbal articulations (i.e., spoken color names), the remaining time after playback was filled with silence to maintain consistent timing across trials. This approach follows the design used by Cinar et al. (2025), where exposure durations were also fixed, but speech content duration varied naturally. The items were followed by a blank screen delay period during which participants had to hold in mind the preceding items, while continuing to perform articulatory suppression. Finally, the probe screen showed a black outline shape with no color fill in the center of the screen, together with a square textbox above it, where participants were to type the color that corresponded to the probed shape in the preceding items. After typing the color name, participants had to press the enter key to submit their response and thereby start the next trial. Each serial position was probed 15 times per binding condition. There were 180 trials in total, amounting to approximately 45 minutes of testing time (not longer than 1h). The opportunity to take 2-minute breaks was offered between each Binding condition, and once halfway through each Binding condition. Feedback on performance was not given.

#### Sampling plan

Data collection followed Bayesian sequential hypothesis testing (Schönbrodt et al., 2017), whereby data were collected in batches of 30 participants until reaching either a Bayes factor (BF) of 10 for or against Confirmatory Hypothesis 2 (that is, the main effect of Binding in accuracy results, not the follow-up t-tests), or a maximal N of 60 participants. The BF was assessed after the initial batch of participants was collected, and their data were cleaned and prepared for analyses. Had the BF not reached 10, another batch of 30 would have been collected and included in the analyses.

#### Data cleaning and analyses

Immediately after data collection, participants’ data were cleaned and made uniform for later processing and analyses, such that writing errors (e.g., *turqoise* instead of *turquoise, 0pink* instead of *pink, purpl* instead of *purple* [translated from French]), and term errors (e.g., *cyan* instead of *turquoise* or *magenta* instead of *pink* when all other colors were recognized correctly [translated from French]) were corrected. When participants’ responses were missing or ambiguous (e.g., writing *I don’t know*, or a new color label when all other possible color labels were reported correctly [translated from French]), their original response was left in the dataset uncorrected, so as not to introduce bias into the data via experimenter guessing. A full list of corrections per participant is present in the Analysis and Results section of the OSF repository for this project (https://osf.io/xkq42/). Next, since all participants completed the experiment in its entirety, accuracy per Binding condition was calculated and the above accuracy-based exclusion criterion was applied, such that participants with accuracy lower than 25% in at least one Binding condition were excluded from analyses. The remaining data were further cleaned by removing trials with reaction times shorter than 200 ms (to remove prepotent responses) or longer than each individual’s average reaction time plus 3 standard deviations (to remove overly long responses reflecting a change of mind during typing).

After the above cleaning procedures, each participant’s mean accuracy was calculated per Serial position per Binding condition. These values were then submitted to a 3 × 4 within-subject repeated measures Bayesian analysis of variance (henceforth BANOVA) with the factors: Binding (UO-V, SS-V, UO-AV) and Serial position (1, 2, 3, 4).

Evidence for the individual terms in the model was assessed through model fit comparisons. Comparing the full model to equivalent models without the effect revealed to what extent the term improves the model fit.^3^ A Bayes Factor of at least 10 meant that there was strong evidence that adding this term improved model fit.

To address Confirmatory hypothesis 1, i.e., the main effect of Serial position, we first planned to assess the evidence for an overall difference between the levels of Serial position. In the event of strong evidence for a main effect (i.e., evidence at or above the BF = 10 threshold), we planned to conduct a post-hoc 1-sided paired Bayesian t-test comparing serial position 1 to serial position 4. We expected the general direction of the effect to follow the pattern from lowest accuracy at serial position 1 to highest accuracy at serial position 4, replicating Cinar and colleagues (2024,4 see also: Jones & Oberauer, 2013; Murdock, 1962). To address Confirmatory hypothesis 2, i.e., the main effect of Binding, we first planned to assess the evidence for an overall difference between the Binding conditions. In the event of evidence at or above the BF = 10 threshold, we planned to conduct two follow-up one-sided paired Bayesian t-tests assessing the exact differences between the levels: One t-test to compare the UO-V and SS-V condition, and the other to compare the UO-AV and UO-V conditions. Here, we expected to replicate Cinar and colleagues (2025) and find evidence for UO-V > SS-V, and evidence against UO-AV > UO-V (specifically, finding evidence for the null, that is, UO-AV = UO-V), respectively.

#### Transparency and Openness

We report how we determined our sample size, all data exclusions, all manipulations, and all measures in the study. All the materials, code, and data are shared in a public OSF repository: https://osf.io/93af2/.

**Figure 3:**
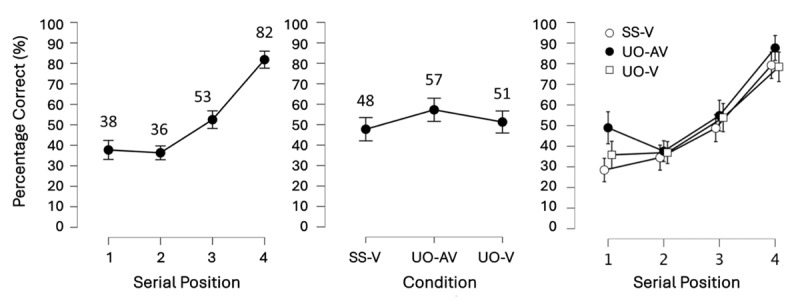
Results of Experiment 1.0 showing the main effects of Serial Position (left) and Binding Condition (center) as well as their interaction (right). Note, however, that the interaction contributed only weakly to the model fit and was thus not further investigated. Mean accuracy (displayed as percent correct) per level of Serial Position and Binding are shown numerically above each point on the graph. Error bars show the 95% credible interval. Serial position increased in the expected pattern, substantiating Confirmatory hypothesis 1. Meanwhile the binding conditions were sufficiently different, producing a main effect of Binding. However, the underlying patterns were unexpected: UO-AV was actually larger than UO-V, and UO-V and SS-V were not meaningfully different from each other.

Data formatting, cleaning, and exclusion criteria were applied using R version 4.0.3 (R Core Team, 2020) running in RStudio v. 2023.09.1 (RStudio Team, 2023), using the libraries: dplyr v.1.0.8 (Wickham, François, Henry, and Müller, 2022), readxl v.1.4.0 (Wickham and Bryan, 2023), openxlsx v.4.2.5 (Schauberger and Walker, 2022), and stringr 1.4.0 (Wickham, 2023). Analyses were conducted using JASP Version 0.18.3 (JASP Team, 2024a), using default settings. To obtain interaction figures (right panel in Figures 3, 5, 7, and 9) JASP Version 0.19.3 (JASP Team, 2024b) was used. The design and analysis of the entire study, except experiment 1.5, were preregistered (available here: https://osf.io/bfpre) after the collection of 30 initial participants for Experiment 1.0, but before their data were cleaned and analyzed.

### Results

We conducted a pre-registered BANOVA with the within-subject factors: Binding (UO-V, SS-V, UO-AV) and Serial position (1, 2, 3, 4). The best model was the model that contained both main effects and their interaction (i.e., the full model). Below we report how we continued from this point in the analysis to address Confirmatory hypotheses 1 and 2. Figure 3 presents mean accuracy data broken down by serial position, by condition, and by serial position within each condition (see supplementary Figure S2 for a visualization of the full data distribution).

#### Confirmatory hypothesis 1: the main effect of Serial position

To assess the contribution of Serial position to the model fit, we first removed the interaction term from the full model – this only made the model 1.63 times worse showing that the interaction did not contribute much to the model. Next, we compared the model with the two main effects to the model containing only the main effect of Binding. This process revealed that Serial position made the model with the two main effects 2.15^e+22^ times better, constituting extremely strong evidence for the main effect of Serial position. A descriptive observation of the means (see Figure 3 left), and a planned follow-up 1-sided paired Bayesian t-test comparing accuracy at serial positions 1 and 4 confirmed that the main effect followed the expected pattern of improvement from serial position 1 to 4. Namely, the t-test showed that accuracy was higher at serial position 4 than at serial position 1, at a BF_–0_ = 2.11^e+10^. Thus, Confirmatory hypothesis 1 was substantiated, and the pattern from Cinar and colleagues (2024) was replicated.

#### Confirmatory hypothesis 2: the main effect of Binding

To assess the contribution of Binding to the model fit, we compared the model with the two main effects to the model containing only the main effect of Serial position, revealing that the factor Binding condition made the model with both main effects 27.81 times better. However, according to the planned follow-up 1-sided paired Bayesian t-tests, the main effect was not produced by the expected differences between the Binding conditions (i.e., UO-V > SS-V, and UO-V = UO-AV, as in Cinar et al, 2025). Instead, we found that accuracy in the UO-V and SS-V conditions were not substantially different from each other (BF_+0_ = 1.13), while accuracy in UO-AV was larger than accuracy in UO-V (BF_+0_ = 13.43; Figure 3, center). Thus, although these results did not replicate those of Cinar and colleagues (2025), they did reveal a bimodal advantage, i.e., more accurate recall for bimodal over unimodal presentation of memoranda.

### Discussion

The accuracy pattern from the first to the fourth serial position replicated Cinar and colleagues (2024) and followed the well-established recency effect in working memory (Glanzer, 1972). In contrast, we did not observe an overall primacy effect.^5^ This differs from findings reported in (forward) serial or free recall paradigms, where large primary effects (and smaller recency effects) are typically reported (reviewed by Oberauer, 2003). These differences are consistent with distinct retrieval demands of these paradigms, which facilitate different mechanisms (Oberauer, 2003). Specifically, recency effects are commonly attributed to input interference or decay during encoding, where later items interfere with earlier ones. Primacy effects in serial recall arise from output interference, when early-recalled items interfere with later items, or from attentional gradients, where early items receive a greater amount of attentional resources. Because the present task tested only a single item per trial, output interference does not play a role, and a pronounced recency effect presents the expected pattern. In fact, this pattern was replicated in subsequent experiments (see Experiment 1.3 – 1.5) and closely aligns with earlier findings in cued recall (Guazzo et al., 2020, Allen et al., 2009, Allen et al., 2006), immediate recognition (Forsberg, Guitard, Greene, Naveh-Benjamin, & Cowan, 2025) and probed recall (Waugh & Norman, 1965), confirming that our task was well-posed to measure working memory recall.

Surprisingly, however, the Binding condition results did not follow our expectations, nor replicate Cinar and colleagues (2025). The lack of difference in recall accuracy observed between the UO-V and SS-V conditions, is perhaps not a serious issue, as a study from the same research group (Allen et al., 2009) preceding that of Cinar and colleagues (2025) has also not found these two conditions to be different. The bimodal advantage, however, was now observed, contrary to a series of previous studies (Allen et al., 2009, Cinar et al., 2025, Guazzo et al., 2020). This was especially surprising given how closely we followed Cinar et al.’s (2025) experimental protocol. To clarify the origins of this surprising bimodal advantage, we conducted Experiment 1.3.

## Experiment 1.3

Having established a bimodal advantage in Experiment 1.0 – contrary to previous work – the aim of Experiment 1.3 was two-fold: First, we aimed to replicate the bimodal advantage under the same conditions as in Experiment 1.0. Second, we aimed to clarify whether the bimodal advantage arises from the actual *storage of a bimodal code* or whether *bimodal presentation* alone is sufficient to boost cued recall performance. If the latter is true, participants may have recoded the auditory (e.g., verbalization of the color “green”) and visual (e.g., the image of a circle) inputs into a common format (e.g., the image of a green circle). To disentangle these possibilities, Experiment 1.3 included two previously tested conditions (UO-V and UO-AV, in the following referred to as UO-AV-P for “presentation”) as well as a novel condition (UO-AV-S for “storage”). In this novel condition, pure tones were used as auditory stimulus material, preventing the formation of a common visual (or verbal) code. This allowed to test whether bimodal storage – beyond bimodal encoding – contributed to the observed advantage.

### Methods

There were many methodological similarities between Experiment 1.0 and Experiment 1.3, and thus, for brevity, the upcoming text will only describe how Experiment 1.3 differed from its predecessor.

#### Participants

A total of 30 young adult University of Geneva undergraduate students participated in Experiment 1.3 in exchange for course credits. Four did not pass the above preregistered exclusion criteria, resulting in a final sample of 26 participants (age range 19–36, mean age 23, 2 males).

#### Design and stimuli

Like Experiment 1.0, Experiment 1.3 had a 3 × 4 repeated-measures factorial design. However, here there was no SS-V condition, but instead, the design contained within-subject factors Condition (Unitized Object-Visual [UO-V], Unitized Object-AudioVisual-Presentation [UO-AV-P], and Unitized Object-AudioVisual-Storage [UO-AV-S]) and Serial position (1, 2, 3, 4). Visual and auditory stimuli were the same as in Experiment 1.0 for conditions UO-V and UO-AV-P. However, in the UO-AV-S condition, auditory stimuli were meaningless tones created in Audacity software v.2.4.2. (Audacity Team, 2020). The tones’ spectral compositions ranged from 100 Hz to 3000 Hz (i.e., 100 Hz, 150 Hz, 220 Hz, 440 Hz, 500 Hz, 900 Hz, 1200 Hz, and 3000 Hz), and they were modulated in terms of their amplitude envelopes and waveform types (square or sinusoid; similar to Thelen et al., 2012, 2014, 2015). All (meaningless) sounds were digitized at 44,100 Hz and presented for 1000 ms. All stimuli used in Experiment 1.3 are available in the Experiment section of the OSF repository for this project (https://osf.io/fg3wb/).

#### Task and procedure

**Figure 4:**
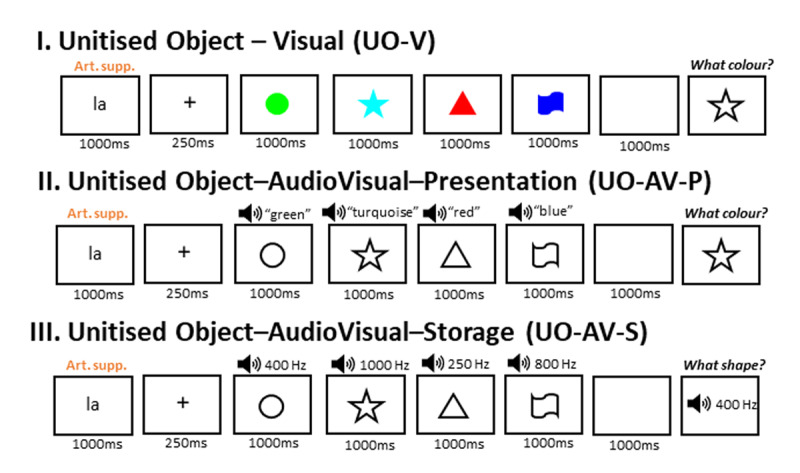
Schematic representation of a trial for each Condition in Experiment 1.3, using the same color-shape combinations as in Figure 2 for comparison purposes. The textbox that participants saw during the probe is omitted from the figure.

The task procedure, trial progression, trial number and experimental duration were the same as in Experiment 1.0. The only difference was that in Experiment 1.3, in the UO-AV-S condition, participants were asked to report the shape that corresponded to a tone in one of the four previously presented shape-tone combinations (see Figure 4). This resulted in a difference with respect to cue feature (color vs. shape) between conditions, which was necessary to allow for a direct replication of the UO-V and UO-AV conditions in Experiment 1.0 (i.e., asking for color based on a shape-cue), while maintaining feasibility of the task with the new stimulus material in the storage condition (UO-AV-S). In the latter, since verbalizing the pitch of a pure tone for a typed response would have been likely impossible (unless explicitly practiced), we intentionally used the pitch as a cue and asked participants to report the associated shape.

#### Sampling plan

The sampling plan was the same as in Experiment 1.0, except that the hypothesis that anchored the stopping rule was Confirmatory Hypothesis 2, i.e., a main effect of Condition (erroneously labelled as Confirmatory hypothesis 1 in the preregistration). That is, data were collected in batches of 30 participants until reaching either a BF = 10 for or against Confirmatory Hypothesis 2, or a maximal N of 60 participants.

#### Data cleaning and analyses

The data cleaning procedure was the same as in Experiment 1.0. Data analyses were also highly similar to Experiment 1.0 in that each participant’s mean accuracy was calculated per Serial position per Condition and then submitted to a 3 × 4 within-subject repeated measures BANOVA with the factors: Binding Condition (UO-V, UO-AV-P, UO-AV-S) and Serial position (1, 2, 3, 4). Confirmatory hypothesis 1, i.e., the main effect of Serial position, was addressed the same way as in Experiment 1.0. Confirmatory hypothesis 2, i.e., the main effect of Condition, was addressed by assessing the evidence for an overall difference between the levels of Condition. In the event of evidence at or above the BF = 10 threshold, and since we arrived at this experiment from Experiment 1.0, we planned to conduct a follow-up one-sided paired Bayesian t-test assessing whether the differences between UO-V and UO-AV-P replicate the pattern found in Experiment 1.0. Specifically, since Experiment 1.0 found strong evidence for an advantage for bimodal presentation over unimodal presentation (UO-AV > UO-V at a BF_+0_ = 13.43), we expected to find such a bimodal advantage here too, shown by strong evidence for UO-AV-P > UO-V.

To address Exploratory hypotheses, we planned to conduct further one-sided paired Bayesian t-tests as follow-ups to the above BANOVA in the event of strong evidence for the main effect of Condition. For Exploratory hypothesis 1, we planned to assess the evidence for UO-AV-S > UO-V, and for UO-AV-S > UO-AV-P, expecting strong evidence for both effects.

### Results

**Figure 5:**
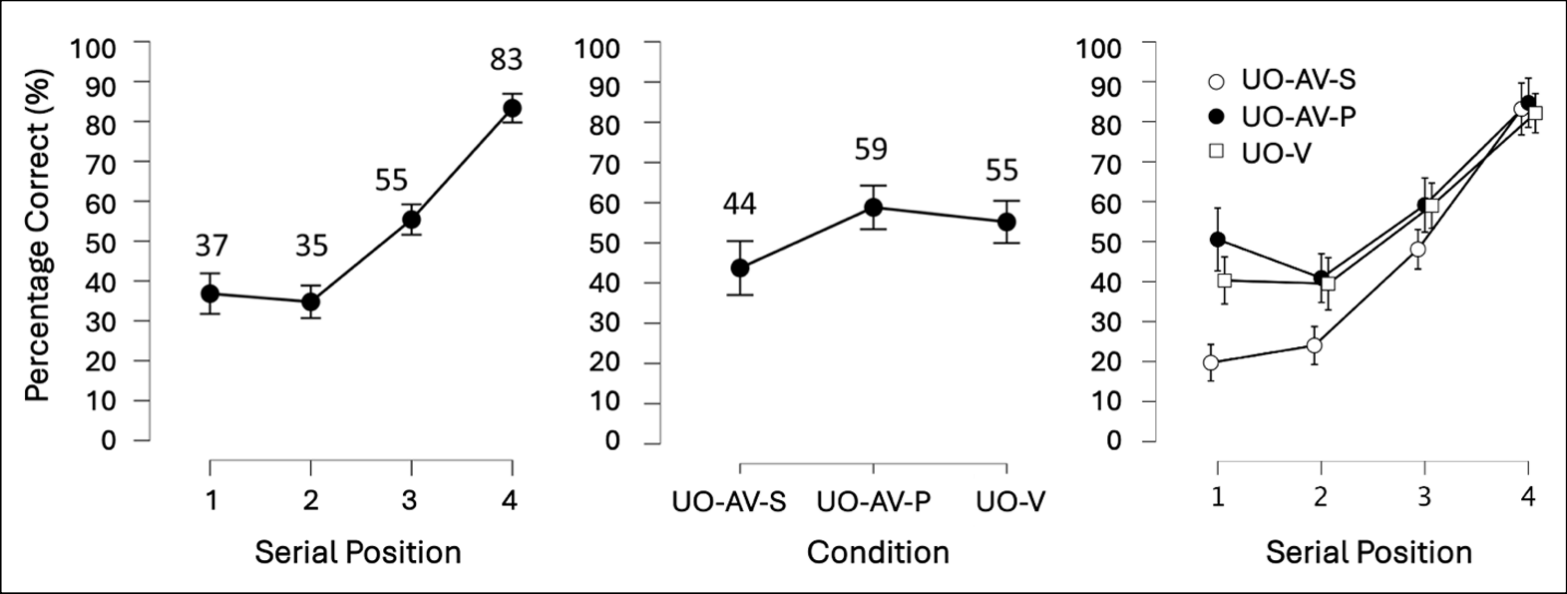
Results of Experiment 1.3 showing the main effects of Serial Position (left) and Binding Condition (center), as well as their interaction (right). Mean accuracy (displayed as percent correct) per level of Serial Position and Binding are shown numerically above each point on the graph. Error bars show the 95% credible interval. Serial position once again increased in the expected pattern, while the levels of Condition once again differed meaningfully, but contrary to expectation. This time, recall accuracy in the UO-AV-S condition was the lowest, drawing out the main effect, and demonstrating what appeared to be a recall cost of bimodal storage. Also, there was no bimodal presentation advantage, as UO-AV-P and UO-V did not differ. Inspecting condition differences at each serial position, however, revealed moderate evidence for a bimodal advantage at serial position 1 in the UO-AV-P condition. In contrast, bimodal costs were evident at serial position 1 to 3 in the UO-AV-S condition.

As in Experiment 1.0, we conducted a pre-registered BANOVA with the within-subject factors: Binding Condition (UO-V, UO-AV-P, UO-AV-S) and Serial position (1, 2, 3, 4). The full model was again the best model, but this time, the interaction also contributed to improving the model fit (removing it made the model 2.09^e+4^ times worse). Thus, as pre-registered, we focused on the main effects of Serial position and Condition to address our Confirmatory and planned Exploratory hypotheses, but the interaction term was examined in an unplanned, exploratory follow-up analysis. Figure 5 presents mean accuracy data broken down by serial position, by condition, and by serial position within each condition (see supplementary Figure S3 for a visualization of the full data distribution).

#### Confirmatory hypothesis 1: the main effect of Serial position

To assess the contribution of Serial position to the model fit, we compared the model with the two main effects to the model containing only the main effect of Condition and found that Serial position made the model with the two main effects 3.38^e+25^ times better. This, as in Experiment 1.0, was extremely strong evidence for the main effect of Serial position, further confirmed by the planned follow-up 1-sided paired Bayesian t-test showing higher accuracy at serial position 4 than at serial position 1, at a BF_–__0_ = 7.59^e+10^ (Figure 5, left). Thus, Confirmatory hypothesis 1 was once again substantiated.

#### Confirmatory hypothesis 2: the main effect of Condition

To assess the contribution of Condition to the model fit, we compared the model with the two main effects to the model containing only the main effect of Serial position, revealing that Condition made the model with both main effects 9.23^e+6^ times better. However, the underlying Condition differences were contrary to expectation. The planned follow-up 1-sided paired Bayesian t-test now revealed only anecdotal evidence for a bimodal advantage, with UO-AV-P and UO-V only being slightly different from each other (Figure 5, centre, BF_+0_ = 2.13). Thus, in Experiment 1.3, we did not substantiate evidence for a bimodal advantage.

#### Exploratory hypotheses: Bimodal costs

Two further follow-up 1-sided paired Bayesian t-tests to the main BANOVA investigated the other Condition differences in an exploratory way since our predictions were less certain as they were not directly based on a previous experiment. We found strong evidence against higher recall accuracy for UO-AV-S than UO-V (BF_0+_ = 22.40), and against higher recall accuracy for UO-AV-S than UO-AV-P (BF_0+_ = 26.15). Indeed, when observing the means per Condition level (Figure 5, center), it is clear that the above t-test results can be explained by accuracy for UO-AV-S being the lowest of all of the Condition levels. To statistically check this observation, we conducted unplanned exploratory 1-sided paired Bayesian t-tests testing whether UO-AV-S < UO-V, and UO-AV-S < UO-AV-P. The results indeed confirmed that UO-AV-S was the lowest-performing condition (UO-AV-S < UO-V, BF_–0_ = 4393.03; UO-AV-S < UO-AV-P, BF_–0_ = 2.20^e+7^). Thus, instead of an added bimodal advantage due to bimodal storage over and above unimodal and bimodal presentation, we observed a bimodal storage cost in recall accuracy.

#### Exploratory hypotheses: interaction of serial position and condition

Following up on the interaction term, which significantly improved model fit, unplanned and non-preregistered individual paired sample t-tests were conducted to assess bimodal advantages (UO-AV-P > UO-V) or costs (UOAVS < UO-V) at each serial position (Figure 5, right). This revealed moderate evidence for a bimodal advantage in the audio-visual presentation condition (UO-AV-P > UO-V) at serial position 1 (BF_+0_ = 4.64), but not at any other position (position 2: BF_+0_ = 0.28, position 3: BF_+0_ = 0.21, position 4: BF_+0_ = 0.46). In contrast, very strong to strong evidence for bimodal costs were observed in the audio-visual storage condition (UO-AV-S < UO-V) at serial positions 1 (BF_–0_ = 5464.45), 2 (BF_–0_ = 56.29), and 3 (BF_–0_ = 16.139), but not at position 4 (BF_–0_ = 0.165).

### Discussion

It was initially surprising to not observe a bimodal presentation advantage in Experiment 1.3 after having detected it in Experiment 1.0, although the analysis revealed a main effect of Condition. However, upon closer investigation, it became clear that descriptively, recall for audio-visually presented features still exceeded performance of all other conditions: yet, the main effect of Condition was primarily a result of low accuracy in the UO-AV-S condition compared to the other two. It is possible that participants had difficulty retaining the meaningless auditory stimuli or that the combination of such meaningless sounds and visual shapes incurred too high of a memory load. Indeed, previous research shows that working memory capacity for pure tones is around 2 to 3 items (Alunni-Menichini et al., 2014; Li et al., 2013; Yuan et al., 2025), and thus lower than typical estimates of 3 to 4 items for simple visual features such as color or shapes (Fukuda et al., 2010; Luck & Vogel, 1997). In addition, participants may have been confused by this condition, as it was the only one that used meaningless sounds and required a shape-based, rather than a color-based, response. Given such potential issues, it was difficult to disentangle whether the observed results were due to a genuine effect of bimodal storage mechanisms differing from bimodal and unimodal presentation mechanisms, or due to this particular condition being disproportionately difficult or cognitively demanding. To minimize the influences of different response formats across conditions, we next conducted Experiment 1.4, per our preregistered plan, as this experiment removes the differences in task settings.

## Experiment 1.4

### Methods

**Figure 6:**
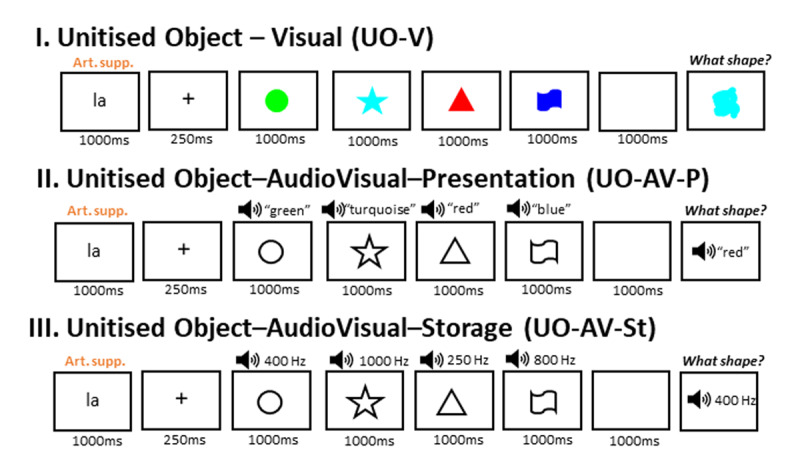
Schematic representation of a trial for each Condition in Experiment 1.4, using the same color-shape combinations as in Figures 2 and 4 for comparison purposes. The textbox that participants saw during the probe is omitted from the figure.

Experiment 1.4 was identical to Experiment 1.3, except for the fact that in *every* Condition, participants were asked to report the shape that corresponded to either the color or sound (color word in UO-AV-P or tone in UO-AV-S) in one of the four previously presented items on that trial (see Figure 6). The data cleaning and analysis plan was also the same as in Experiment 1.3. In Experiment 1.4, a total of 60 young adult University of Geneva undergraduate students participated in exchange for course credits. Eight did not pass the above preregistered exclusion criteria, resulting in a final sample of 52 participants (age range 19 – 36, mean age 22, 13 males).

### Results

**Figure 7:**
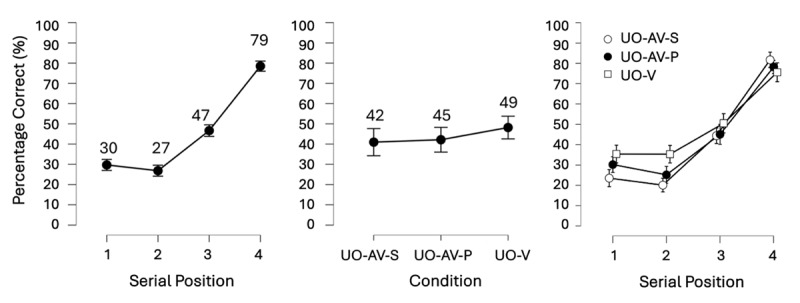
Results of Experiment 1.4 showing the main effects of Serial Position (left), Binding Condition (center), and their respective interaction (right). Mean accuracy (displayed as percent correct) per level of Serial Position and Binding are shown numerically above each point on the graph. Error bars show the 95% credible interval. Serial position again increased according to expectations. The levels of Condition once again differed meaningfully, such that recall accuracy for UO-V was highest, while recall accuracy for the audiovisual conditions (i.e., UO-AV-P and UO-AV-S), which did not differ from each other, was lower. Thus, there was no bimodal advantage, but rather a bimodal cost, if anything. Comparisons at each serial position mostly corroborated the pattern of bimodal costs, except at the last serial position, at which moderate evidence for a bimodal advantage (UO-AV-S > UO-V) was observed.

As in Experiment 1.3, we conducted a pre-registered BANOVA with the within-subject factors: Condition (UO-V, UO-AV-P, UO-AV-S) and Serial position (1, 2, 3, 4). The full model was once again the best model. Removing the interaction term made the model 4.88^e+4^ times worse. Thus, as pre-registered, we focused on the main effects of Serial position and Condition, but the interaction term was examined in an exploratory follow-up analysis. Figure 7 presents mean accuracy data broken down by serial position, by condition, and by serial position within each condition (see supplementary Figure S4 for a visualization of the full data distribution).

#### Confirmatory hypothesis 1: the main effect of Serial position

As in both previous experiments, comparing the model with the two main effects to the model containing only the main effect of Condition showed extremely strong evidence for the main effect of Serial position by revealing that Serial position made the model with the two main effects 6.37^e+58^ times better. The follow-up 1-sided paired Bayesian t-test confirmed this by showing higher accuracy at serial position 4 than at serial position 1, at a BF_–0_ = 6.34^e+25^ (Figure 7, left). Thus, Confirmatory hypothesis 1 was once again substantiated.

#### Confirmatory hypothesis 2: the main effect of Condition

As in both previous experiments, comparing the model with the two main effects to the model containing only the main effect of Serial position, revealed very strong evidence for the main effect of Condition, as Condition made the model with both main effects 85.65 times better. However, once again, the underlying Condition differences were not according to expectations. There was again no bimodal advantage, as the planned follow-up 1-sided paired Bayesian t-test revealed strong evidence against accuracy in UO-AV-P being larger than in UO-V (Figure 7, right, BF_0+_ = 24.79). Therefore, if anything, there seems to have been a bimodal cost.

#### Exploratory hypotheses: bimodal costs

A series of further follow-up 1-sided paired Bayesian t-tests to the main BANOVA confirmed the bimodal cost suspicion. First, planned exploratory t-tests revealed strong evidence against higher recall accuracy for UO-AV-S than UO-V (BF_0+_ = 30.75), and against higher recall accuracy for UO-AV-S than UO-AV-P (BF_0+_ = 17.10). Unplanned t-tests then assessed whether, as the Condition level means would suggest (Figure 7, right), UOV was actually the highest performing condition. This was confirmed by 1-sided paired Bayesian t-tests showing that UO-V accuracy was larger than UO-AV-P accuracy (BF_0+_ = 16.73), and UO-AV-S accuracy (BF_0+_ = 365.67). The final check for a bimodal cost or unimodal advantage was a t-test comparing whether UO-AV-P accuracy was larger than UO-AV-S accuracy, and it was not (BF_0+_ = 1.96). Thus, recall in the two audiovisual conditions was similar, with an improvement in the visual-only condition.

#### Exploratory analyses: interaction of serial position and condition

A series of one-sided paired sample-tests (UO-AV-P > UO-V) revealed moderate to strong evidence against a bimodal advantage at serial positions 1–3 (position 1: BF_+0_ = 0.06, position 2: BF_+0_ = 0.03, position 3: BF_+0_ = 0.06) and anecdotal evidence against a bimodal advantage at serial position 4 (BF_+0_ = 0.466). Very strong evidence for bimodal costs in the audio-visual storage condition compared to the visual condition (UO-AV-S < UO-V) was evident at positions 1 (BF_–0_ = 288.03) and 2 (BF_–0_ = 76635.04) but remained anecdotal at serial position 3 (BF_–0_ = 1.42). At serial position 4, strong evidence against bimodal costs was observed (BF_–0_ = 0.05), and indeed, the reversed directional test (UO-AV-S > UO-V), revealed moderate evidence for a bimodal advantage (UO-AV-S > UO-V) at serial position 4 (BF = 3.38).

### Discussion

Now that participants had to report the same information (shape) in all conditions, UO-AV-S was no longer the worst-performing condition. Indeed, as opposed to Experiment 1.3 where 2 of the 4 excluded participants were removed for failing to understand the UO-AV-S condition (they appear to have thought they needed to recall all of the shapes they saw on that trial), in Experiment 1.4, exclusions showed no such consistent patterns. Thus, given that in Experiment 1.4 task rules were balanced across conditions, we assume that the results reflected genuine cognitive processes. However, there was still no overall bimodal advantage, not even for bimodal presentation, and let alone for bimodal storage in working memory. Only upon closer examination of the interaction term, a bimodal advantage emerged at serial position 4 for the audiovisual storage over the unitized visual presentation condition. The lack of a consistent bimodal advantage is in strong contrast with the results of Experiment 1.0. Further, in Experiment 1.3 observing the means (Figure 5) suggested that the advantage could have been detected if the bimodal storage condition had not been overly difficult compared to the other conditions, artificially creating strong evidence for a main effect of Condition. There are two potential reasons as to why we did not observe an advantage for bimodal presentation in Experiment 1.4, both of which relate to probe type (i.e., in Experiments 1.0 and 1.3, participants had to recall colors cued by shape [or pitch] probes, whereas in Experiment 1.4 they had to recall shapes cued by color [or pitch]). First, it is possible that recalling shapes (in Experiment 1.4) is more difficult than recalling colors (in Experiments 1.3, and 1.0). Previous studies have shown that shape is recalled slightly less accurately than color (Allen et al., 2012; Schneegans et al., 2023). However, why this would affect the presence of a bimodal advantage on recall, mechanistically speaking, is not clear. Shape and color seem like interchangeable stimulus features for working memory studies such as the present one – both are frequent in daily life, and both are codable both visually and verbally. Second, it is possible that in the bimodal presentation condition of Experiment 1.4, articulatory suppression impoverished participants’ color representations, since they were presented aurally as words. This may have affected encoding or subsequent storage of those features, which in turn may have hindered recall when color was used to cue shape recall. In fact, previous studies have shown that swap errors in cued recall studies can be explained by variability in memory for the cue feature (McMaster, Tomic, Schneegans & Bays, 2022). In Experiments 1.3, and 1.0, articulatory suppression would have also affected phonological representations of color, but intact shape cues could have allowed for easier access to the linked representations of colors, even if the latter were potentially less precise. Thus, it could be that, when the feature that was presented verbally in the bimodal presentation condition is used to cue recall, performance is worse than when the visually presented feature is used to cue recall in the same condition. To test this hypothesis, in an attempt to pin down the task settings supporting the bimodal advantage, we conducted Experiment 1.5. It included one unisensory and two bimodal conditions, differing in whether the verbally or visually presented feature was used as the retrieval cue.

## Experiment 1.5

### Methods

Experiment 1.5 was conceived as a follow-up to Experiment 1.4, so we will only describe aspects that differed from its predecessor. It was preregistered after the preceding experiments were concluded, and after an initial round of data collection (25 participants) and data cleaning (i.e., correction of spelling and term errors as in the previous experiments). The preregistration is available at: https://osf.io/7hm8d. To briefly summarize: we preregistered that we would conduct analyses as in Experiment 1.4 once the preregistration was frozen, and that, regardless of the results at that stage, we would collect additional data to arrive at 30 participants. Then, if at this stage we did not achieve strong evidence for or against Confirmatory hypothesis 2 (i.e., main effect of condition), we would keep collecting data until we did, or until we reached the maximal N of 60 participants. In a slight deviation from this preregistration, we did *collect* the full dataset (i.e., up to an N of 60 participants) in a single batch following the preregistration, due to organizational constraints. However, in line with the preregistration, we only analyzed the data of the first 5 participants to construct the first batch of 30 participants and then analyzed the remaining data.

#### Participants

A total of 62 young adult University of Geneva undergraduate students participated in Experiment 1.5 in exchange for course credits. The number of participants tested ended up being slightly higher than our planned maximal N of 60 due to compensation for no-shows. Four participants did not pass the above preregistered exclusion criteria, resulting in a final sample of 58 participants (age range 17 – 65, mean age 24, 15 males).

#### Design and stimuli

**Figure 8:**
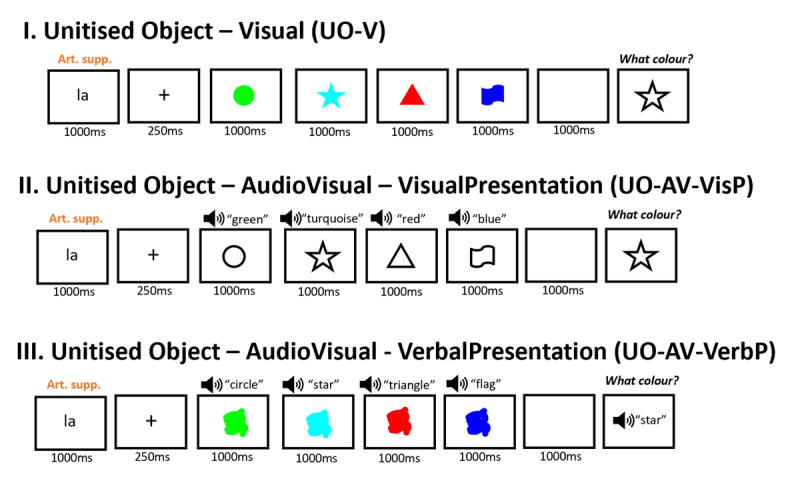
Schematic representation of a trial for each Condition in Experiment 1.5, using the same color-shape combinations as in the previous figures for comparison purposes. The textbox that participants saw during the probe is omitted from the figure.

Like the previous experiments, Experiment 1.5 had a 3 × 4 repeated-measures factorial design, now with within-subject factors Condition (Unitized Object-Visual [UO-V], Unitized Object-AudioVisual-VisualPresentation [UO-AV-VisP], and Unitized Object-AudioVisual-VerbalPresentation [UO-AV-VerbP]) and Serial position (1, 2, 3, 4). Importantly for this experiment, participants always engaged in color recall, which was cued in different ways across the different conditions: with a visual shape stimulus in conditions UO-V and UO-AV-VisP, and with a verbal shape word in the UO-AV-VerbP condition (see Figure 8). The UO-AV-VisP condition was the same as the UO-AV-P condition in Experiment 1.3 (weak evidence towards a bimodal advantage) and the UO-AV condition in Experiment 1.0 (revealed a bimodal advantage). Meanwhile, the UO-AV-VerbP condition was the same as the UO-AV-P condition in Experiment 1.4 (revealed no bimodal advantage). The auditory stimuli used in the UO-AV-VerbP condition had the same parameters as other verbal stimuli in this and previous experiments. Specifically, they were recordings (digitized at 44,100 Hz) of the same female native French speaker uttering the names of the eight shapes otherwise presented visually in this and the previous experiment, in French, set to a volume of 13 (on a 0-100 scale) on each testing computer. All stimuli are available in the Experiment section of the OSF repository for this project (https://osf.io/fg3wb/).

#### Task and procedure

The task procedure, trial progression, trial number and experimental duration were the same as in Experiments 1.3 and 1.4. The only difference was that in Experiment 1.5, participants always had to report the color of a previously presented item (see Figure 8).

#### Sampling plan

Initially, we collected data from 25 participants and, after preregistering our analysis plan, used this data to examine preliminary results. We then tested 37 additional participants. The data were analyzed in two batches (see justification above): Based on the analysis of the first 30 participants, we assessed whether the evidence for or against Confirmatory Hypothesis 2 (a main effect of condition) reached a Bayes Factor (BF_10_ or BF_01_) of at least 10, as specified in our preregistered stopping rule. Since the evidence remained inconclusive at this stage, we added the remaining data into the sample, resulting in 58 participants analyzed (after exlusions).

#### Data cleaning and analyses

The data cleaning and analysis procedures were the same as in Experiment 1.4, except that the 3 × 4 within-subject repeated measures BANOVA contained the factors: Condition (UO-V, UO-AV-VisP, UO-AV-VerbP) and Serial position (1, 2, 3, 4). In the event of evidence at or above the BF=10 threshold for the main effect of Condition, we planned to conduct two follow-up one-sided paired Bayesian t-tests assessing: (a) whether recall accuracy in the UO-AV-VisP condition was larger than in the UO-V condition, constituting a bimodal advantage (as seen in Experiment 1.0 and with weak evidence in Experiment 1.3) and (b) whether recall accuracy in the UO-AV-VerbP condition was smaller than in the UO-V condition, as seen in Experiment 1.4 with the analogous AV-P and UO-V conditions. We expected the analyses to confirm this pattern, based on our results from previous experiments.

### Results

**Figure 9:**
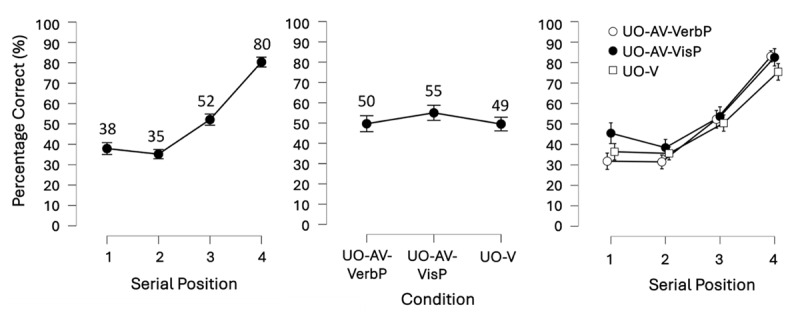
Results of Experiment 1.5 showing the main effects of Serial Position (left) and Binding Condition (middle) as well as their interaction (right). Mean accuracy (displayed as percent correct) per level of Serial Position and Binding are shown numerically above each point on the graph. Error bars show the 95% credible interval. Serial position once again increased according to expectations. Recall accuracy for UO-AV-VisP was higher than UO-V, showing evidence for a bimodal advantage when the recall cue was visual. However, there was no difference between the UO-AV-VerbP and UO-V conditions, showing no bimodal advantage for verbal recall cues. Follow-up tests, resolving the interaction, revealed strong evidence for a bimodal advantage at serial position 1 and 4 with visual probe presentation (UO-AV-VisP > UO-V). With verbal cue presentation, recall accuracy was diminished or comparable for audiovisual compared to unisensory trials (UO-AV-VerbP < UO-V) at serial positions 1 to 3, showing evidence against a bimodal advantage. At serial position 4, however, recall performance in the UO-AV-VerbP exceeded that in unisensory trials, showing very strong evidence for a bimodal advantage with verbal cues.

As in prior experiments, the pre-registered BANOVA with the within-subject factors Condition (UO-V, UO-AV-P, UO-AV-S) and Serial position (1, 2, 3, 4) showed that the full model was the best model, and the interaction improved the model fit (removing it made the model 90.9 times worse). As before, we focused on the main effects of Serial position and Condition below but examined the interaction in an exploratory follow-up analysis. Figure 9 presents mean accuracy data broken down by serial position, by condition, and by serial position within each condition (see supplementary Figure S5 for a visualization of the full data distribution).

#### Confirmatory hypothesis 1: the main effect of Serial position

As before, there was strong evidence for the main effect of Serial position: Comparing the model with the two main effects to the model containing only the main effect of Condition revealed that Serial position made the model with the two main effects 7.57^e+55^ times better. The follow-up 1-sided paired Bayesian t-test confirmed this and thus substantiated Confirmatory hypothesis 1 by showing higher accuracy at serial position 4 than at serial position 1, at a BF_-0_ = 4.68^e+22^ (Figure 8, left).

#### Confirmatory hypothesis 2: the main effect of Condition

As in previous experiments, there was strong evidence for the main effect of Condition, as comparing the model with the two main effects to the model containing only the main effect of Serial position revealed that Condition made the model with both main effects 37.58 times better. As for the underlying Condition differences, now there was evidence for a bimodal advantage in the visual presentation condition (UO-AV-VisP > UOV, BF_+0_ = 223.48), but not in the verbal presentation condition (UOV > UO-AV-VerbP, BF_0+_ = 7.66). Indeed, upon visual inspection (Figure 8, right), recall accuracies in the UOV and UO-AV-VerbP conditions seem almost equal.

### Exploratory hypotheses: the interaction of serial position and condition

A series of one-sided paired-sample t-tests examined the bimodal advantage at each serial position. With visual probe presentation, strong evidence for a bimodal advantage (UO-AV-VisP > UO-V) was observed at serial position 1 (BF_+0_ = 8.34) and serial position 4 (BF_+0_ = 22.52). In contrast, at serial position 2 (BF_+0_ = 0.45) and 3 (BF_+0_ = 0.43) evidence for or against a bimodal advantage remained inconclusive. With verbal probe presentation, moderate to strong evidence against a bimodal advantage (UO-AV-VerbP > UO-V) was observed at serial positions 1 to 3 (position 1: BF+0 = 0.05, position 2: BF+0 = 0.05, position 3: 0.26). Surprisingly, at serial position 4 very strong evidence for a bimodal advantage emerged (BF+0 = 162.43), in line with the descriptive data showing better performance in the UO-AV-VerbP compared to the UO-V condition (see Figure 8, right panel).

### Discussion

With Experiment 1.5, we now have two full experiments (1.0 and 1.5) demonstrating strong evidence for a bimodal advantage in cued recall task settings, as well as a third experiment (1.3) providing weak evidence. Experiment 1.5 confirms that the bimodal advantage emerges with a visual recall cue prompting recall of color information. An exploratory examination of the interaction term further revealed that the bimodal advantage is primarily driven by performance benefits for items at the first and the last serial position. While the unplanned nature of these follow-up analyses warrants caution, it suggests a special role for items in the focus of attention (Morisson et al. 2014). In contrast, recall in the condition with a verbal recall cue for color information was not different from recall in the unimodal visual condition. That is, the bimodal advantage was not observable when a verbal recall cue triggered recall of color information, except for the last serial position – which again confirmed a bimodal advantage. Taken together, these results support the robustness of the bimodal advantage, while qualifying the task settings in which it can be observed. Namely, the presentation format of the probe seems to be an important contributor to the bimodal advantage, as the results confirmed our expectation that recall is worse when the feature with a more fragile representational format is used as the cue (due to task settings like articulatory suppression), compared to when the more robustly represented feature serves as the cue.

## General Discussion

Motivated by a discrepancy in the working memory literature regarding the task conditions that give rise to a bimodal advantage – a supposedly general phenomenon –, the current study investigated the bimodal advantage in a cued recall task. Across four experiments, we clarify the specific circumstances under which a bimodal advantage occurs, offering a deeper understanding of this perceptuo-cognitive phenomenon. Below, we summarize our findings and discuss the results in relation to prior studies that did not find a bimodal advantage, potential mechanisms underlying the effect, and the broader framework of the multicomponent model of working memory.

First, across all experiments, we observed the expected recency effect curve (Glanzer, 1972), showing that our tasks were well-posed to measure working memory recall. Second, employing near-identical task settings to previous studies (Cinar et al. 2025, Guazzo et al 2020), and in contrast with this literature, we found evidence for a bimodal advantage across multiple experiments, suggesting that the effect is more robust than previously assumed. Third, we found that audiovisual presentation alone was sufficient to produce the bimodal advantage. Conversely, additional task manipulations designed to promote bimodal storage (beyond bimodal encoding, see UO-AV-S condition in Experiment 1.3 and 1.4) did not enhance – and in fact failed to elicit – the bimodal advantage. Notably, these manipulations likely increased task difficulty to the point of impairing performance, which may have obscured the effect. Together, these results suggest that the bimodal advantage arises out of perceptual-level audiovisual enhancement, likely taking place when memoranda are encoded. Fourth, we ascertained that the bimodal advantage disappeared when the cue feature has a more fragile representation, namely, when the cue constitutes a feature that was presumably encoded or stored less robustly due to interference from articulatory suppression. Finally, an exploratory examination of the interaction between serial position and condition in experiments 1.3, 1.4. and 1.5, suggested that the bimodal advantage may be particularly prone to emerge for items that have received extended rehearsal or still reside in the focus of attention (i.e., the first and/or the last item).

### Comparing Present and Prior Findings on the Bimodal Advantage in Cued Recall

First and foremost, it is striking that we find robust evidence for a bimodal advantage across several experiments, while previous studies using nearly identical task-settings did not (Allen et al., 2009, Cinar et al., 2025, Guazzo et al., 2020). However, several methodological and analytical differences may account for this discrepancy.

A first factor concerns differences in task and response demands: The original study by Allen and colleagues (2009) used the same task conditions and stimulus timing as our Experiment 1.0, but with a smaller set size (three items) and a slightly different response format: they always presented a visually unitized color-shape combination as a probe and asked participants to indicate whether it was present or absent in the memorized sequence. Although the use of lures (i.e., a recombination of features from different items) ensured that the task required feature binding, this response format may have encouraged visual encoding strategies, thereby reducing the relative benefit of cross-modal presentation. Moreover, binary match versus no-match decisions likely depend more strongly on familiarity and thus may involve different cognitive mechanisms than cued recall, which requires more precise, feature-specific retrieval.

Second, inconsistent findings may arise because previous studies disregarded the effect of cue type. In a cued recall paradigm, Guazzo et al. (2020) randomly varied from trial to trial whether participants had to report a (visually presented) shape based on a (verbally presented) color cue or vice versa, but did not analyze potential interactions with cue dimension. In contrast, our results show that the bimodal advantage is more robust when the visual feature (which is encoded without interference from articulatory suppression) is used as a recall cue, while this effect does not arise when the auditory feature dimension is used as a cue. Thus, disregarding this factor may have masked or attenuated the effect in previous studies.

Third, mixed evidence may arise due to interactions with serial position. Our exploratory analyses in Experiments 1.3 – 1.5 demonstrate that the bimodal advantage is most pronounced at the first or last serial position. This implies that the effect primarily arises for items which reside in the focus of attention or have undergone extended rehearsal. This resembles findings in the *modality effect* (reviewed Penney, 1989) and *production effect* literature (Saint-Aubin, Yearsley, Poirier, Cyr, & Guitard, 2021, Gionet, Guitard, & Saint-Aubin, 2022), where auditory or produced items (i.e., items read aloud at encoding) show a mnemonic advantage over visually presented (and silently read) items. Notably, this effect is typically restricted to the last (or the last two) serial positions. However, prior cued recall studies have rarely considered such interactions. When serial position was examined, analyses focused on position-wise accuracy differences rather than testing whether condition differences varied by position (Guazzo et al., 2020) or effects observed at individual serial positions were reported, but not further considered (Cinar et al., 2024, *preprint*^6^). As a consequence, previous studies likely overlooked potential interactions with serial position. Indeed, both of these studies contain indications consistent with a bimodal advantage. Guazzo et al, for instance, note descriptively higher accuracy in the cross-modal compared to the unimodal condition among older adults in their second experiment. In addition, Cinar et al. (2024) observed a significant bimodal advantage in two of three experiments at the last serial position. However, given that neither study had the primary objective to detect a bimodal advantage, these patterns were not further explored in their original reports.

Lastly, all of the above studies included smaller sample sizes between 16 and 35 participants, with either no sample size rationale (Allen et al., 2006), or with power analyses geared towards other manipulations (e.g., reward prioritization effects or age group differences; Cinar et al. 2025; Guazzo et al. 2020). In contrast, our pre-registered sequential testing procedure allowed us to collect data until strong evidence (BF > 10) for a bimodal advantage emerged or a maximum sample size of 60 was reached. While Experiment 1.0 showed a robust bimodal advantage with a sample of 28 participants — comparable to the above studies — Experiment 1.5 required data from 58 participants, suggesting that larger samples may be critical to reliably detect the effect. Taken together, subtle differences in design, task format, and analytic focus may explain why the present study was better positioned to reliably detect a bimodal advantage.

#### Bimodal presentation versus bimodal storage

Upon initially demonstrating a bimodal advantage in experiment 1.0, we set out to differentiate whether the bimodal advantage would rest on the mere existence of multiple input modalities or the maintenance of information as a multimodal code. A range of previous studies has shown that the representational format of objects in working memory does not necessarily correspond to the sensory presentation format (Conrad, 1964; Guitard & Cowan, 2020; Logie, Della Salla, Wynn & Baddeley, 2000). For instance, basic sensory features, such as color, may be recoded and maintained as in an action-oriented motoric code if a given response format can be anticipated (Bae & Chen, 2024, Henderson et al., 2022). Moreover, physically distinct visual features (such as orientations and moving random dot patterns) can be retained in a common abstract format (Kwak & Curtis, 2022). This introduces the possibility that bimodal presentation is not necessarily followed by bimodal maintenance.

To assess the contribution from bimodal storage (beyond the role of bimodal presentation), Experiment 1.3 and 1.4 introduced a condition in which pure tones were used as auditory input (cf. UO-AV-S condition). The latter cannot be easily verbalized or visualized and thus required that they were retained in their original acoustic format. Likewise, visual shapes cannot be recoded into a meaningful acoustic code. Thus, a bimodal advantage in this condition could only arise from actual bimodal storage. Contrary to what we anticipated, however, the condition involving pure tones proved much more difficult than the conditions involving verbalizable stimuli (i.e., colors and shapes). In consequence, not only was there no bimodal advantage in those conditions, but instead bimodal costs occurred. Notably, Experiment 1.4 ruled out that this cost was due to discrepancies in recall format between different task conditions (shape recall vs. color recall). This points to an alternative explanation: namely, that with pure tones as stimulus material, participant’s working memory capacity was exceeded. In fact, working memory capacity for simple pure tones is on average 2 to 3 items (Alunni-Menichini et al., 2014; Li et al., 2013; Yuan et al., 2025), while experiments with simple visual features such as shape and color typically reach capacity estimates of 3 to 4 items (Fukuda et al., 2010; Luck & Vogel, 1997). Consequently, combining pure tones with visual shapes (or colors) may have posed a greater load on a modality-general storage capacity such as the episodic buffer (Baddeley, 2000), thereby eliminating any potential benefit from bimodal storage.

Taken together, the present data do not speak to a storage-based account and instead suggest that the bimodal advantage arises during perceptual encoding. However, it seems premature to rule out contributions from bimodal storage entirely. Indeed, Experiment 1.4, with all task settings equalized, revealed a bimodal advantage for combinations of pure tones and shapes over combinations of shapes and colors at the last serial position. This suggests that, when within the focus of attention, pure tones were generally capable of eliciting a bimodal advantage.

In line with the importance of cross-domain processing during the initial formation of binding, Allen et al. (2015) showed that spatial tapping during encoding abolished the benefit from combining verbal and spatial information in a digit recall task. In contrast, it did not eliminate the cross-domain benefit when executed exclusively during recall. The authors argued that the observed cross-domain advantage must therefore result from the formation of a domain-general representation during encoding, and not solely from participants being able to draw on modality-specific representations at recall. They further argued that such domain-general representations are subsequentially *retained in a modality-general store*. However, given that their paradigm investigated immediate recall, it remains difficult to disentangle the formation of a cross-modal representation from their subsequent storage. Similarly, in our own design, the serial presentation format complicates a clear distinction between encoding and maintenance, as participants encode later items while maintaining earlier ones.

An alternative explanation for the lack of a bimodal advantage in the conditions with pure tones may lie in the absence of a meaningful semantic association between the auditory and visual inputs. In contrast to verbal colors and shapes, which are more easily integrated conceptually (e.g., “green circle”), the pairing of arbitrary tones and shapes may not have supported strong associative binding. We return to the role of semantic coherence in cross-modal integration in the section on mechanisms below.

Taken together, our findings emphasize that presentation format plays a critical role in shaping working memory performance, particularly in cross-modal contexts. While recent work has focused on representational formats in which information is maintained, our results suggest that the modalities through which information is initially presented continue to exert meaningful influence. This echoes early research on the modality effect (e.g., Crowder & Morton, 1969; see also Penney, 1975, 1989), which highlighted performance differences arising from input modality.

While the present data do not provide strong evidence for a storage-based account of the bimodal advantage, particularly in the case of semantically sparse inputs like pure tones, contributions from bimodal storage cannot be decisively ruled out. Subtle effects at specific serial positions indicate that under certain conditions—such as within the focus of attention—bimodal storage may still confer benefits. Accordingly, future studies may benefit from reducing set size when using difficult-to-recode stimuli such as pure tones, and from incorporating EEG or MEG to help disentangle the temporal dynamics of encoding and maintenance in serial presentation paradigms.

### Mechanisms underlying the bimodal advantage

In the following, we turn to the mechanisms that may give rise to the bimodal advantage in our experiments (see also Mastroberardino et al., 2008 for an extensive review of possible mechanisms). The bimodal advantage has been linked to the activation of both verbal and nonverbal codes (Johnson et al., 1996; Thompson & Paivio, 1994) and both visual and auditory processing streams (Penney, 1989, p. 402-403), as opposed to a single code or processing stream for unimodal presentation. Thus, presenting object features through different sensory channels may result in richer representations, that ultimately increase the distinctiveness of working memory contents (Nairne, 1990). According to the feature model (Nairne, 1990; Neath, 2000), such a “*complex multiattribute memory trace*” (Nairne, 1990, p. 252) contains both the physical, modality-dependent features as well as internally generated modality-independent features in the form of a phonological, semantic or imaginal code. The observation that multiple forms of representational code – established through multimodal encoding or internal recording – lead to an enhancement of working memory performance is not limited to the case of multiple sensory formats, but also extends to broader use cases, such as motoric representations in memory for instructions (Allen et al., 2023) or visuospatial bootstrapping (i.e., support of verbal memory through visuo-spatial memory) in digit recall (Allen et al., 2015; Darling et al., 2017).

However, it is critical to note that the mere stimulation of multiple input channels does not appear to be sufficient to elicit a bimodal advantage. In serial recall studies, such as Delogu et al. (2009), the bimodal advantage emerged when participants were presented with semantically congruent, but complementary audio-visual features (e.g., an image of a dog and the corresponding sound of a dog barking). This was the case, even though the task could in principle be performed by relying on one modality only (e.g., the sound of barking as well as the image of a dog both tell me that the object I am supposed to remember is a dog). On the other hand, the bimodal advantage was absent when participants received redundant information through both visual and auditory channels (i.e., the printed and spoken word “dog”). In line with the Feature Model, this emphasizes that the bimodal benefit rests on the availability of non-redundant information through both input channels. In our paradigm, auditory and visual features were not only non-redundant, but uniquely informative – such that both input modalities were essential in reconstructing the memorized item (e.g. a visual shape + a spoken color). Thus, successful recall required participants to integrate these features into a unified multimodal representation.

Alternatively, participants may have recoded both visual and auditory information into a common code (e.g., the image of a “green circle”). Imagery-based recoding or the generating of a common verbal code^7^ (e.g., green circle) could also encourage deeper, more elaborative processing – which would likely not be present with fully redundant input (such as the concurrent presentation of a printed and a spoken word). This raises the possibility that the bimodal advantage results from stronger engagement with the memoranda rather than multimodality per se. However, if this was the case, the presentation of two visual, spatially segregated features should have incurred a similar depth of processing if participants internally generated a unified representation from those inputs. While this seems to contradict the elaborative processing hypotheses, it remains to be considered that in the real word, visual features of the same object are rarely spatially segregated. Thus, from a multicomponent model point of view, integrating spatially segregated visual features may have placed a greater load on the central executive (i.e., involving strategic combination of features) than the binding of cross-modal or visually unitized features, which are presumed to enter the episodic buffer rather passively (Baddeley et al., 2011; Hitch et al., 2025). This, in turn, could explain the behavioral cost observed in the spatially segregated visual condition (SS-V, Exp 1).

The lack of a bimodal advantage Experiment 1.3 and 1.4 with pure tones as auditory stimulus material further raises the possibility that meaningful semantic links between the two inputs contributes to the bimodal advantage. Consistently, Delogu et al. (2009) previously argued that the bimodal advantage may only emerge when semantic memory supports the formation of meaningful associations between otherwise unrelated auditory and visual inputs. In line with this proposal, recognition memory for visual objects has been shown to be better when they were paired with object-congruent sounds at encoding, but not when the latter where incongruent (Heikkilä et al., 2015, 2017).

Yet, ERP research suggests that cross-modal integration occurs even with meaningless or arbitrarily paired low-level stimuli, such as tactile stimulation and white noise bursts (Murray et al., 2005) or visual flashes and simple sounds (Giard & Peronnet, 1999; Talsma & Woldorff, 2005). These findings suggest that – at least on a perceptual level – bottom-up integration may still take place, even without higher-order conceptual links. This would be in line with the idea of rather passive and automatic access to a cross-domain episodic buffer, as proposed in the multicomponent model of working memory (Baddeley et al., 2011). It remains possible, however, that semantic links support subsequent maintenance and serve as an effective scaffold for later retrieval. Accordingly, memory performance for low-level features such as color has been shown to improve when those features are encoded as part of meaningful objects, even the identity of the latter is fully task-irrelevant (Chung et al., 2023).

In sum, several candidate mechanisms have been proposed to account for the bimodal advantage. Theories emphasizing multimodal coding, semantic congruence, and elaborative processing all converge on the idea that richer encoding leads to more effective memory traces.

### Is the bimodal advantage mainly driven by the modality effect?

The modality effect (reviewed by Penney, 1989) refers to a robust mnemonic advantage for auditorily presented items at the end of a list in serial recall tasks. This advantage is thought to arise because auditory stimulus presentation, as opposed to visual stimulus presentation, elicits an additional acoustic code. The latter was originally thought to reside in a pre-categorical acoustic store that holds an echoic, sensory memory trace of the most recent item (Crowder & Morton, 1969). However, later accounts, such as the Feature Model (Nairne, 1990; Saint-Aubin et al., 2021), have expanded on this theory. Specifically, the Feature Model distinguishes two types of features: *Modality-dependent* features refer to sensory-based traces that arise as a result of physical stimulus presentation and perceptual processing. In addition, *modality-independent* features are generated through internal processes such as categorization or identification. Critically, auditorily presented information is proposed to include a greater number of modality-dependent features. Further, the model posits that at the end of a list, an internally generated process overwrites modality-independent features, while modality-dependent features remain largely intact. In consequence, recall of auditory items at the last serial position is thought to benefit from the greater number of these modality-dependent features. This raises the question whether the bimodal advantage observed here – in particular at the last serial position – could reflect this classical phenomenon.

Several factors suggest that a modality effect alone cannot account for the observed results: First, the present task required cross-modal binding, such that successful performance rests on accurate memory of both the visual and auditory features, and moreover, the binding between the two. Thus, superior memory for auditorily presented features cannot, by itself, enhance performance, unless the visual cue reliably activates the correctly bound feature. Thus, a modality effect cannot fully explain the asymmetric pattern we observed.

In addition, binding of non-redundant auditory and visual features has been shown to be accompanied by non-linear cross-modal interactions at a neural level (Fort et al., 2002), indicating that the resulting representation is not merely the sum of two independent sensory codes. Such integrative processing strongly suggests that encoding in the present task setting engaged mechanisms that extend beyond a simple auditory superiority account. Future neuroimaging work may help clarify how the behavioral bimodal advantage observed here relates to neural signatures of cross-modal integration.

Finally, we observed a bimodal advantage not only at the final serial position, but also for the first item in two of the four experiments. Such primacy effects are not predicted by the modality effect literature, and suggest that additional mechanisms, such as selective prioritization, contributed to the observed bimodal advantage. This would be in line with attentional gradient accounts of primacy effects (Brown, Preece & Hulme, 2000; Farrell & Lewandowsky, 2002, Lewandowsky & Murdock, 1989, Page & Norris, 1998). According to the latter, items will receive progressively fewer attentional resources during encoding, the later they appear in a list, yielding an activation gradient across serial positions. Bimodal stimuli could amplify this effect by capturing attention more effectively at list onset, producing a selective bimodal advantage at position one.

In sum, while a classical modality effect may have contributed to performance, in particular at the last serial position, we conclude that the observed pattern cannot be fully accounted for by superior memory for auditory information.

### The bimodal advantage through the lens of the multicomponent model of working memory

The above accounts focus primarily on the encoding stage and provide limited insight into how bimodal representations are maintained, updated, or retrieved in working memory tasks involving sequential presentation and cued recall. To address this, we now turn to the multicomponent model of working memory (Baddeley & Hitch, 1974; Hitch et al., 2025), which offers a structural account of how cross-modal information may be bound, maintained, and retrieved.

Notably, the task we adapted from Allen et al. (2009) and Cinar et al. (2025) was itself developed within the framework of the multicomponent model and thus is well suited to probing interactions between modality-specific and modality-general storage processes. The model proposes the episodic buffer as the central structure responsible for binding features across different input modalities. While the initial framework assumed that binding was attentionally demanding, requiring the central executive, the model was later revised (Baddeley et al., 2011), granting the separate slave systems (i.e., the visuo-spatial sketchpad or the phonological loop) direct access to the buffer. In their most recent reflection on how empirical findings have shaped the model (Hitch et al., 2025), Hitch and colleagues equate the episodic buffer with the current focus of attention, “comprising a limited number of integrated, bound representations, their identity influenced by both perceptual and internal control processes” (p. 228). They further note that “the latter [may] reflect task set[s] in the form of goals and plans.” This perspective is interesting as it accounts for our findings, showing that the bimodal advantage appears to emerge primarily for the first or the last item in the sequence. That the last item would still reside in the focus of attention seems intuitively plausible, as it was the last one to be encoded. That the first item may have received strategic prioritization – such that it resided in the episodic buffer longer or more often – also aligns with anecdotal evidence of participants reporting to focus on the first item in a series, despite all of them being equally relevant (Hitch, Allen, Baddeley, 2025).

The model also allows us to explain the inconsistent pattern of results, depending on which feature was used as a cue, by assuming that domain-specific processes (i.e., storage within the phonological loop) are still critical to performance in our cued recall paradigm. Specifically, items outside the current focus of attention are thought to fragment into their separate features within the individual domain-specific buffers (Hitch et al., 2025). Supporting this view, Allen et al. (2015) found that taxing spatial processing resources during encoding abolished the previously observed benefits of cross-domain storage. On a similar note, our results showed that when the verbally encoded feature was used as a recall cue, no bimodal advantage emerged. This aligns with the idea that concurrent articulatory suppression interfered with encoding and subsequent storage in the phonological buffer – reinforcing the central role of domain-specific processing in our task.

## Conclusion

The broader aim of this study was to advance our understanding of how working memory operates in a sensorily rich and complex world. Most everyday environments involve information presented across multiple modalities, yet laboratory studies have historically focused on unimodal inputs, often reducing memory tasks to isolated verbal or visual streams. Our findings contribute to a growing body of evidence suggesting that this approach underestimates the role of cross-modal processing in working memory.

Across four experiments, we demonstrate that a bimodal advantage in working memory can reliably emerge, but only under specific task conditions. The advantage seems to arise primarily at encoding, though we cannot decisively rule out contributions from bimodal storage. Yet, these findings imply that in real-world settings, where input is often multimodal and semantically rich, working memory may routinely leverage inputs from different sensory channels to support flexible and robust performance. More broadly, these results challenge us to think of working memory not as a set of isolated sensory buffers, but as a dynamic, context-sensitive system that flexibly integrates information across modalities to meet the demands of everyday cognition.

## Data Accessibility Statement

All the materials, code, and data are shared in the following public OSF repository, https://osf.io/93af2/. The study design, hypotheses, and methods of the first 3 experiments were preregistered at https://osf.io/bfpre. An additional 4^th^ experiment was conducted, with preliminary data collected and cleaned, followed by a preregistration for continued data collection and analyses regardless of the results (more detail in manuscript), here https://osf.io/7hm8d. This version of the manuscript has been deposited as a preprint to the PsyArXiv repository here: https://osf.io/preprints/psyarxiv/7j6yt_v2.

## Additional File

The additional file for this article can be found as follows:

10.5334/joc.481.s1 Supplementary Materials. The supplementary material file contains the full study flowchart, additional figures for all experiments with individual participant data points, and a supplementary analysis of primacy and recency effects.

## Footnotes

Note that at the time of pre-registration, the study of Cinar and colleagues was not available as a pre-print yet. The authors kindly shared a copy of their conference poster (Cinar et al., 2023) as well as a preliminary version of their manuscript’s methods section with us, to facilitate our efforts to replicate their task design.

To transform hue values into degrees on the color wheel, apply the following formula: hue * (360/256). Thus, for example, the degree for yellow is 42 * (360/256) = 59, which is just 1 degree short of the central value for yellow as defined on the HSL color wheel.

Note that reported BFs for individual model terms are not always equivalent to the BF_incl_ in the shared jasp files. The reported BFs were manually calculated based on the BF in the model comparison section. For instance, to determine to what extend the interaction term improved model fit in experiment 1, the BF of the full model (i.e., the best model) was divided by the BF for the model including the two main effects (i.e., 1 / 0.612 = 1.63).

Note that we refer to the preprint version of Cinar et al., 2025 here in which performance was still reported per serial position. These analyses were removed from the revised, published manuscript.

Note that our preregistered hypotheses only included a test of the first versus the last serial position. To fully differentiate primacy from recency effects, we conducted a supplementary analysis contrasting performance for primacy and recency items relative to middle-position items (see supplementary material, section 3)

Note that this refers to an earlier version of Cinar et al., 2025 available as a preprint on OSF (Cinar et al., 2024; https://doi.org/10.31234/osf.io/59fnw), which differs in the presented analyses from the published manuscript. Originally, Cinar et al. had reported a significant interaction between binding type and serial position. The respective follow-up t-tests, did confirm a significant bimodal advantage at the last serial position.

Note that in the present study, articulatory suppression should have prevented re-coding the visual information into a verbal code.
