# Supplementary material for: Presenting Features Audiovisually Improves Working Memory for Bindings
Source: J Cogn. 2026 Jan 27;9(1):12. doi: 10.5334/joc.481 (PMC12857621; doi:10.5334/joc.481)
Supplement: Supplementary Materials. — The supplementary material file contains the full study flowchart, additional figures for all experiments with individual participant data points, and a supplementary analysis of primacy and recency effects. [file joc-9-1-481-s1.pdf]

SUPPLEMENTARY MATERIALS FOR

Presenting features audiovisually improves working memory for bindings

Nora Turoman[1,2], Elodie Walter[1], Anaë Motz[1], Laura-Isabelle Klatt[3]

[1] Faculty of Psychology and Educational Sciences, University of Geneva, Geneva, Switzerland

[2] Center for the Interdisciplinary Study of Gerontology and Vulnerabilities, University of Geneva, Carouge, Switzerland

[3] Leibniz Research Centre for Working Environment and Human Factors, Dortmund, Germany

Nora Turoman: https://orcid.org/0000-0003-2655-8259; email: nora.turoman@unige.ch

Elodie Walter: https://orcid.org/0009-0002-1111-0208; email: elodie.walter74@gmail.com

Anaë Motz: email: anae.motz@etu.unige.ch

Laura-Isabelle Klatt*: https://orcid.org/0000-0002-5682-5824; email: laura-isabelle.klatt@donders.ru.nl

*Corresponding author

1. Full study flowchart

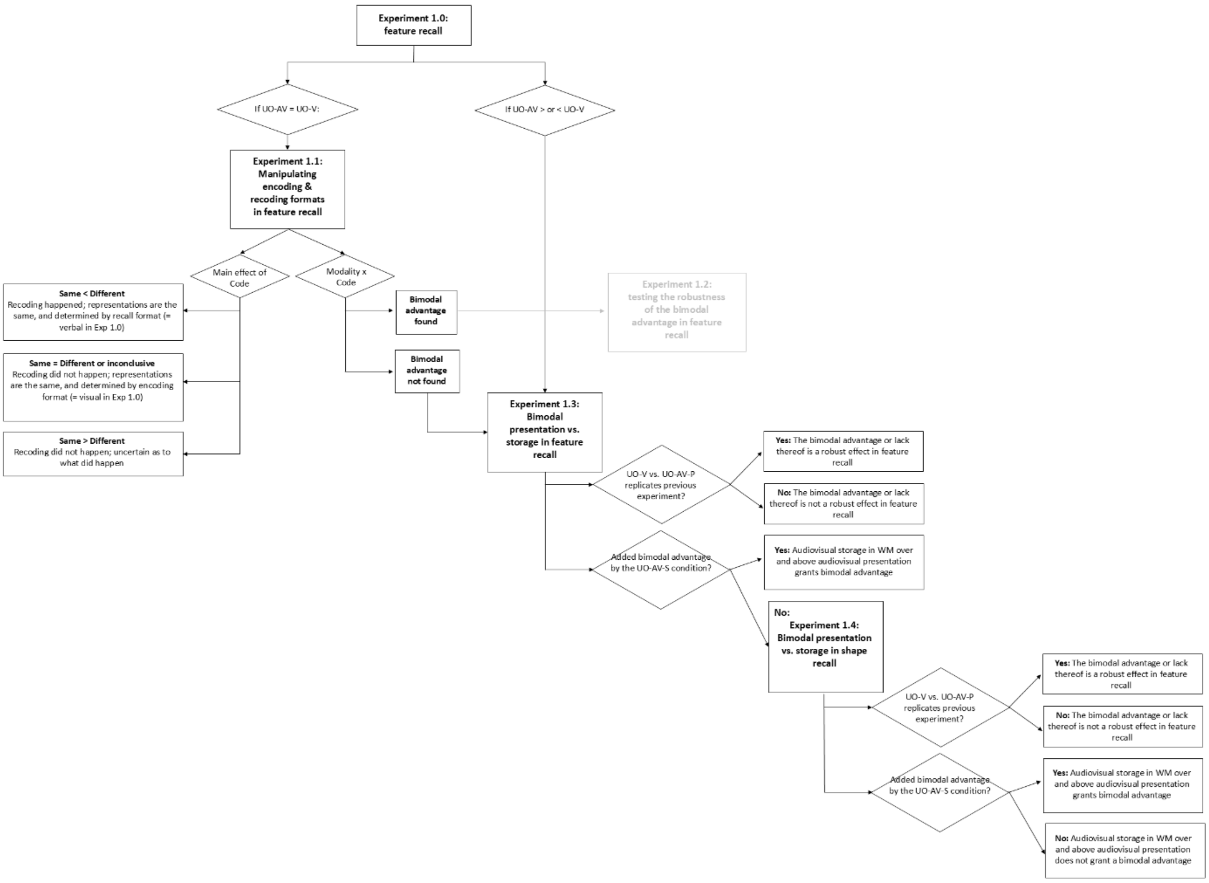

Figure S1. A flowchart detailing the planned experiments following the initial experiment (Experiment 1.0). Experiments were carried out sequentially (top to bottom), depending on the decision taken after the preceding experiment – Experiments and results are shown in square boxes, and decisions are shown in diamond boxes. Experiments that were actually conducted are highlighted in green.

2. Supplementary Visualization of Participant-Level Data

While the figures in the main manuscript illustrate mean accuracy values with 95% credible intervals, the following supplementary figures provide a more detailed view of the data distribution. Data were visualized using the *daviolinplot* function (Karvelis, 2020).

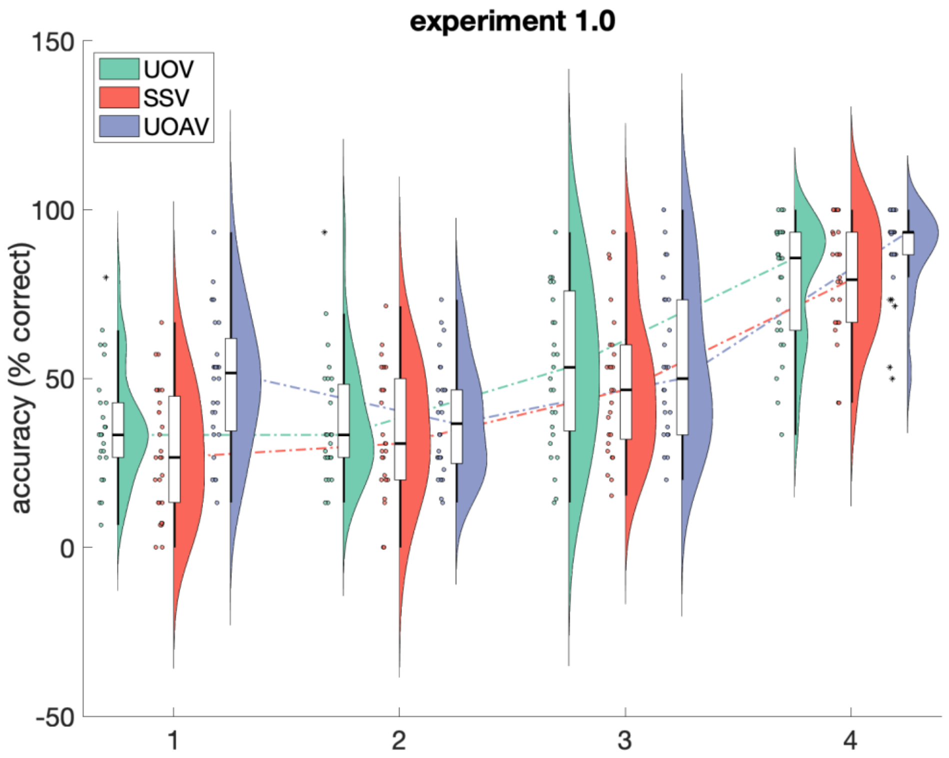

Figure S2. Accuracy (percentage correct) within conditions and at each serial position in experiment 1.0. UOV = Unitised Object – Visual. presentation. SSV = Spatially Separated Object. UOAV = Unitised Object – Audiovisual. X-axis labels 1 to 4 correspond to the respective serial positions. Violin plots depict kernel density estimates of the data distribution, scaled for comparability across conditions. Boxplots within each violin represent the interquartile range (25th–75th percentile), with horizontal lines indicating the median. Whiskers extend to the most extreme values within 1.5 times the interquartile range; outliers beyond this range are plotted individually. Scattered dots represent individual data points, jittered for visibility.

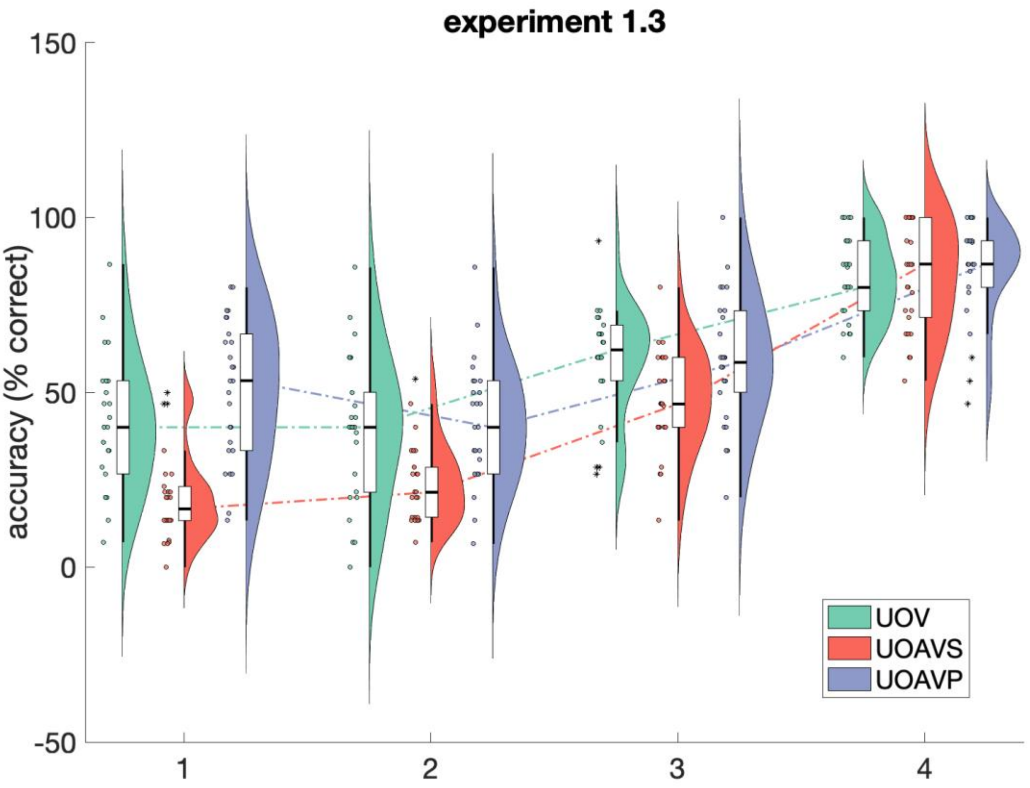

Figure S3. Accuracy (percentage correct) within conditions and at each serial position in experiment 1.3. UOV = Unitised Object – Visual. presentation. SSV = Spatially Separated Object. UOAV = Unitised Object – Audiovisual. X-axis labels 1 to 4 correspond to the respective serial positions. Violin plots depict kernel density estimates of the data distribution, scaled for comparability across conditions. Boxplots within each violin represent the interquartile range (25th–75th percentile), with horizontal lines indicating the median. Whiskers extend to the most extreme values within 1.5 times the interquartile range; outliers beyond this range are plotted individually. Scattered dots represent individual data points, jittered for visibility.

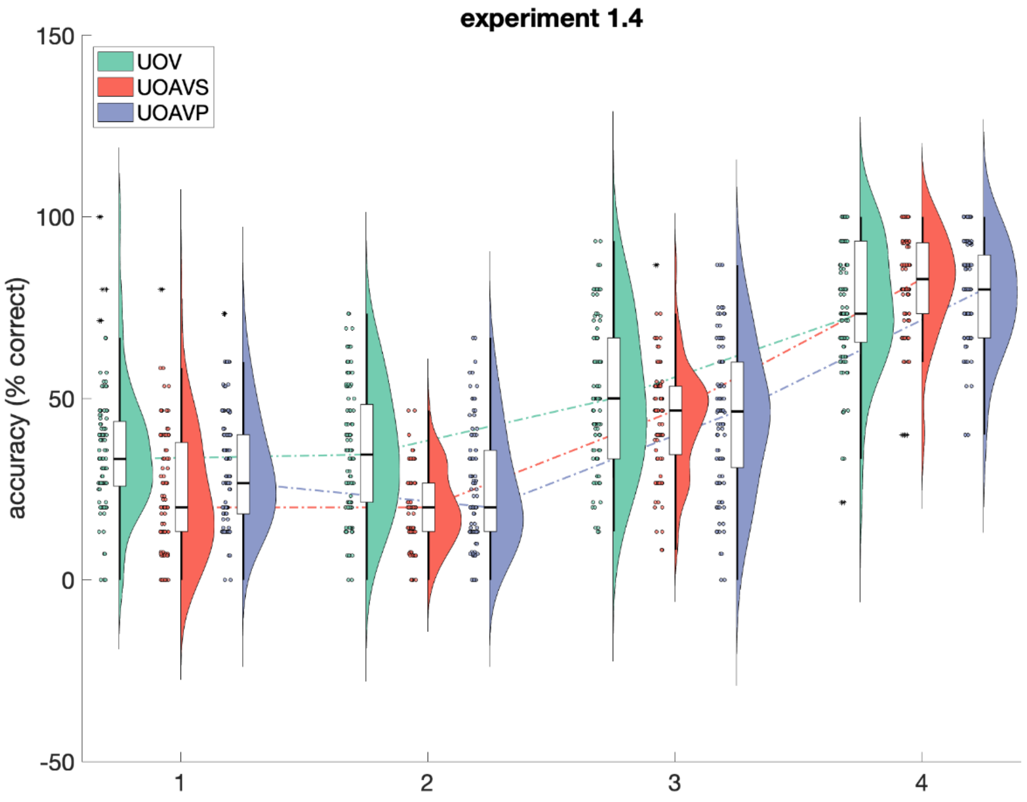

Figure S4. Accuracy (percentage correct) within conditions and at each serial position in experiment 1.4. UOV = Unitised Object – Visual. presentation. SSV = Spatially Separated Object. UOAV = Unitised Object – Audiovisual. X-axis labels 1 to 4 correspond to the respective serial positions. Violin plots depict kernel density estimates of the data distribution, scaled for comparability across conditions. Boxplots within each violin represent the interquartile range (25th–75th percentile), with horizontal lines indicating the median. Whiskers extend to the most extreme values within 1.5 times the interquartile range; outliers beyond this range are plotted individually. Scattered dots represent individual data points, jittered for visibility.

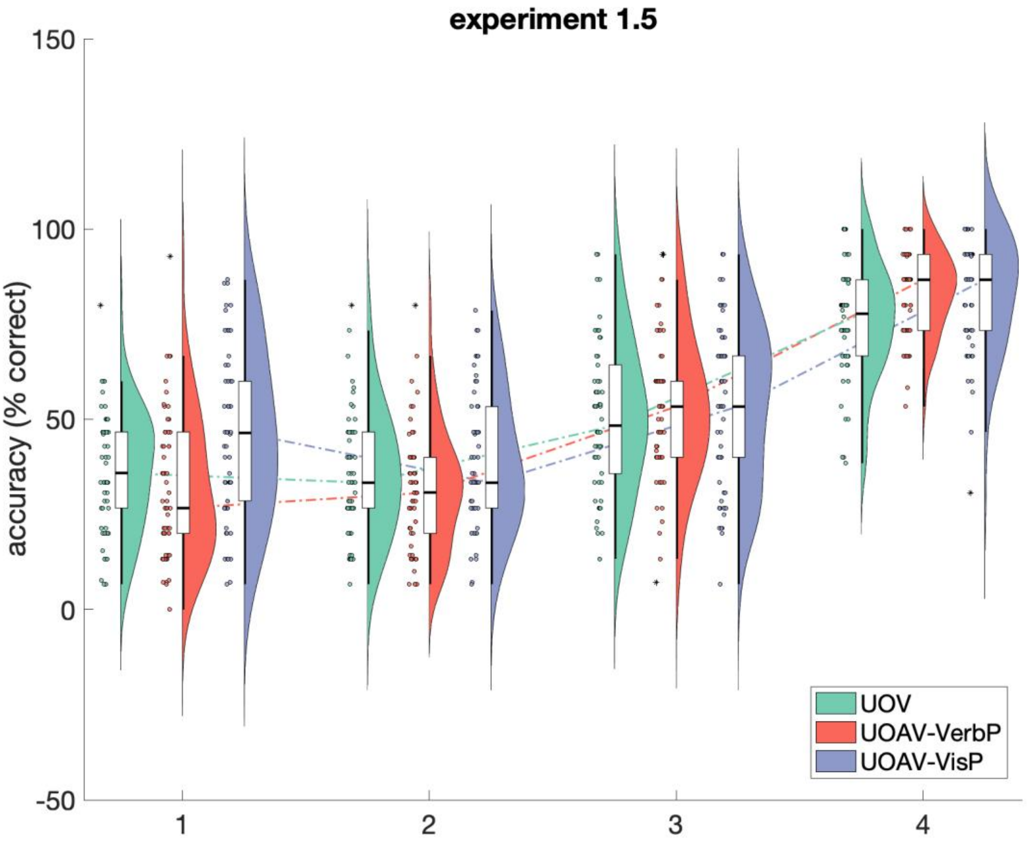

Figure S5. *Accuracy (percentage correct) within conditions and at each serial position in experiment 1.5. UOV = Unitised Object – Visual. presentation. SSV = Spatially Separated Object. UOAV = Unitised Object – Audiovisual. X-axis labels 1 to 4 correspond to the respective serial positions.* Violin plots depict kernel density estimates of the data distribution, scaled for comparability across conditions. Boxplots within each violin represent the interquartile range (25th–75th percentile), with horizontal lines indicating the median. Whiskers extend to the most extreme values within 1.5 times the interquartile range; outliers beyond this range are plotted individually. Scattered dots represent individual data points, jittered for visibility.

3. Analyses of recency and primacy effects

Our preregistered analysis plan included only a direct test of performance between serial position 1 and 4. To better contextualize the serial position effects with the primacy and recency effect literature, we provide a direct test with intermediate positions below. All conducted Bayesian paired sample t-tests were one-sided, assuming an effect in the expected direction (i.e., 1 > 2 and 1 > 3 for primacy effects and 4 >2 and 4 > 3 for recency effects). In sum, we find strong evidence for a recency effect but no evidence for a primacy effect.

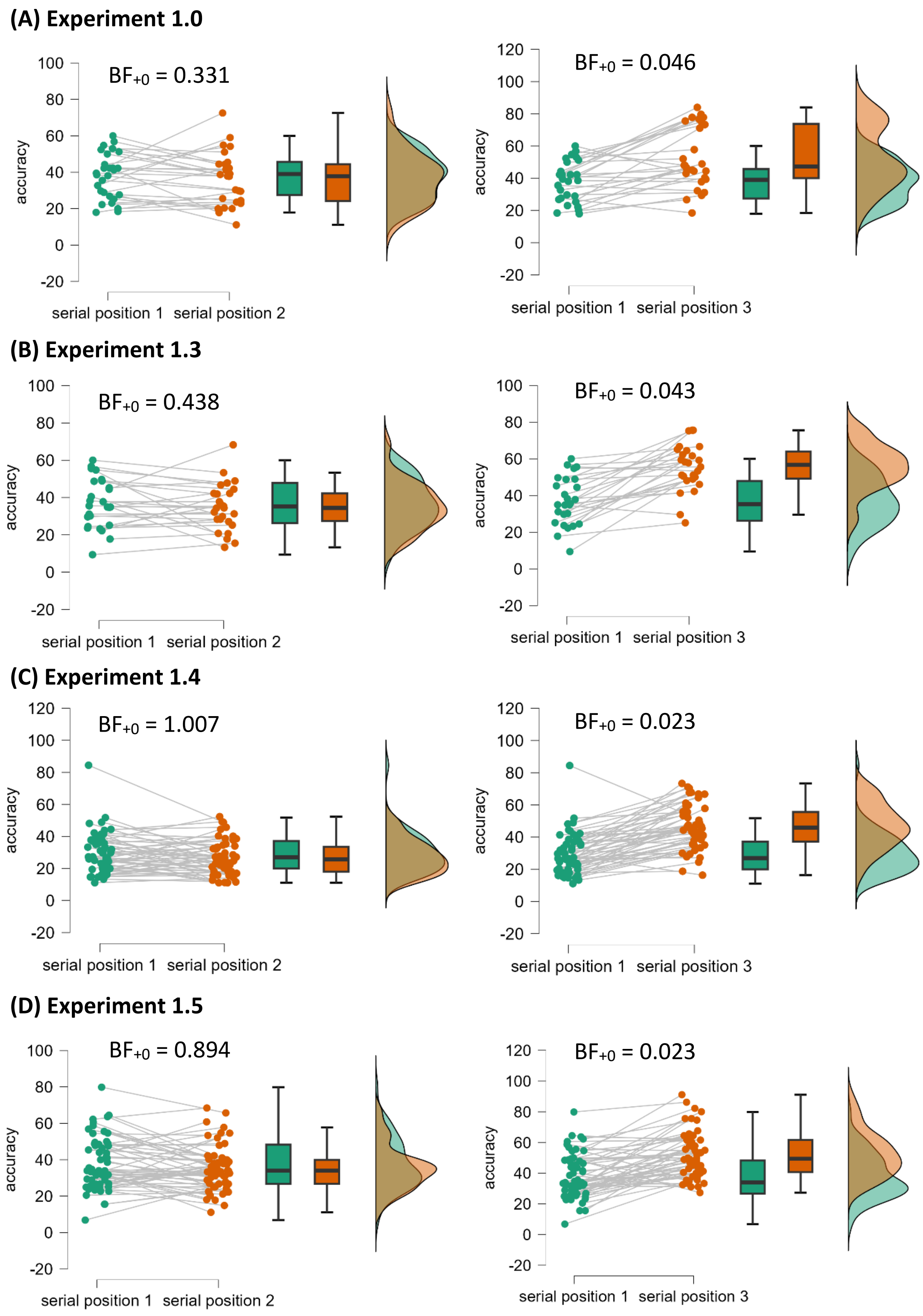

Figure S6. Direct test of primacy effects in all four experiments. Panels A – D show that across all experiments, there is weak evidence for a general primacy effect.

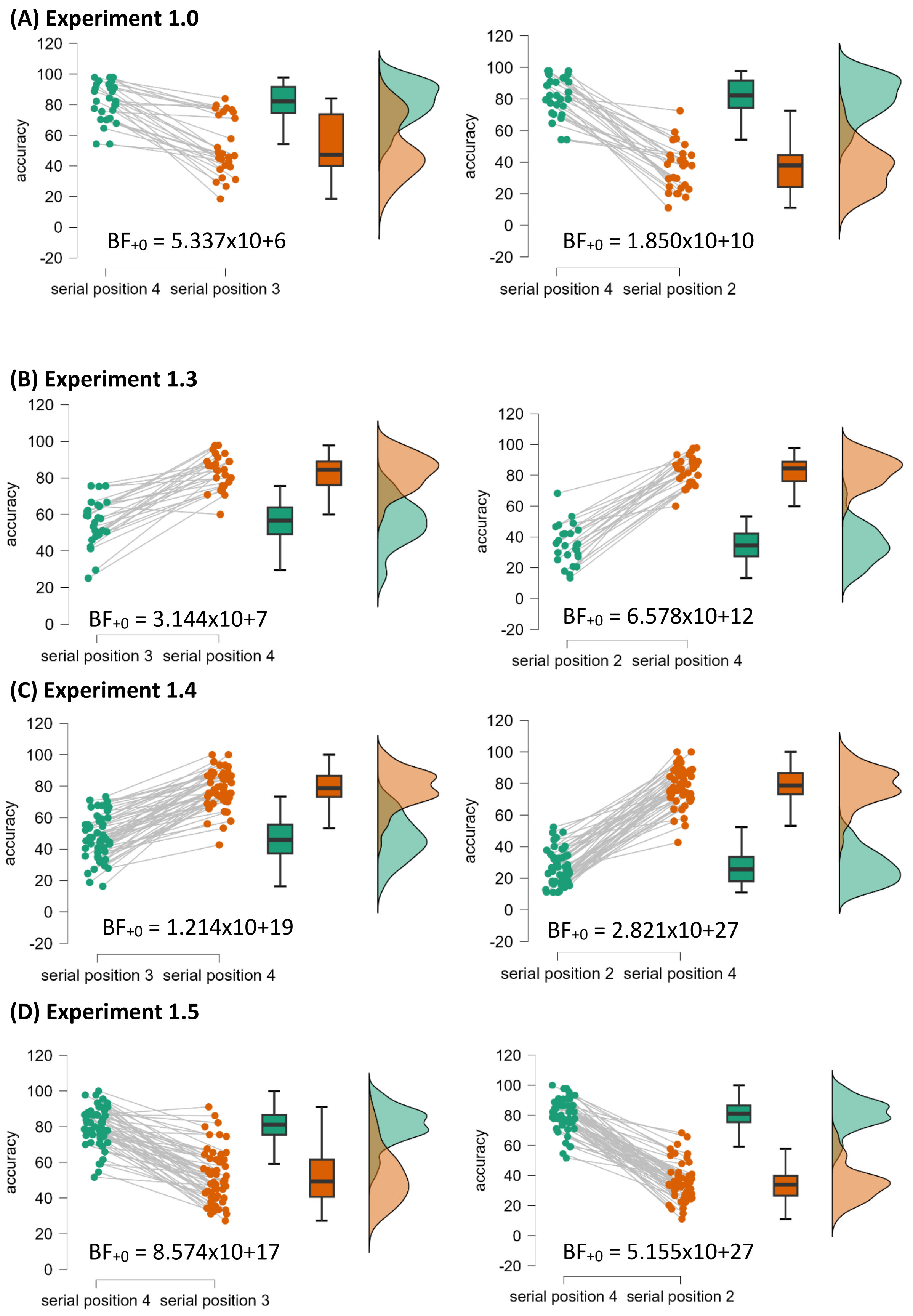

Figure 1. Direct test of recency effects in all four experiments. Panels A – D show that across experiments, there is strong evidence for a recency effect with better performance at the last serial position.

REFERENCES

Karvelis, P. (2020). *daviolinplot: Violin and raincloud plots* (MATLAB Central File Exchange). https://www.mathworks.com/matlabcentral/fileexchange/136524-daviolinplot-violin-and-raincloud-plots
